# Maraviroc as Intensification Strategy in HIV-1 Positive Patients with Deficient Immunological Response: an Italian Randomized Clinical Trial

**DOI:** 10.1371/journal.pone.0080157

**Published:** 2013-11-14

**Authors:** Stefano Rusconi, Paola Vitiello, Fulvio Adorni, Elisa Colella, Emanuele Focà, Amedeo Capetti, Paola Meraviglia, Clara Abeli, Stefano Bonora, Marco D’Annunzio, Antonio Di Biagio, Massimo Di Pietro, Luca Butini, Giancarlo Orofino, Manuela Colafigli, Gabriella d’Ettorre, Daniela Francisci, Giustino Parruti, Alessandro Soria, Anna Rita Buonomini, Chiara Tommasi, Silvia Mosti, Francesca Bai, Silvia Di Nardo Stuppino, Manuela Morosi, Marco Montano, Pamela Tau, Esther Merlini, Giulia Marchetti

**Affiliations:** 1 Divisione Clinicizzata di Malattie Infettive, DIBIC “Luigi Sacco”, Università degli Studi, Milano, Italy; 2 ITB-CNR - Segrate (MI), Italy; 3 Clinica di Malattie Infettive e Tropicali, Università degli Studi, Brescia, Italy; 4 I Divisione di Malattie Infettive, Ospedale Luigi Sacco, Milano, Italy; 5 II Divisione di Malattie Infettive, Ospedale Luigi Sacco, Milano, Italy; 6 Divisione di Malattie Infettive, Ospedale di Circolo, Busto Arsizio (VA), Italy; 7 Clinica delle Malattie Infettive, Ospedale Amedeo di Savoia, Università degli Studi, Torino, Italy; 8 Clinica di Malattie Infettive, A.O.-Universitaria Policlinico, Bari, Italy; 9 Clinica delle Malattie Infettive, Ospedale San Martino, Università degli Studi, Genova, Italy; 10 Divisione di Malattie Infettive, Ospedale S. Maria Annunziata, Antella, Firenze, Italy; 11 Servizio di Immunologia Clinica e Tipizzazione. Tissutale, A.O.-Universitaria, Torrette di Ancona, Italy; 12 Divisione A di Malattie Infettive, Ospedale Amedeo di Savoia, Torino, Italy; 13 Istituto di clinica Delle Malattie Infettive, Università Cattolica del Sacro Cuore, Roma, Italy; 14 U.O. Malattie Infettive, Università La Sapienza, Policlinico Umberto I, Roma, Italy; 15 Clinica delle Malattie Infettive, Policlinico Monteluce, Perugia, Italy; 16 Divisione Clinicizzata di Malattie Infettive, Ospedale Santo Spirito, Pescara, Italy; 17 Divisione Clinicizzata di Malattie Infettive, Ospedale san Gerardo, Monza, Italy; 18 Clinica delle Malattie Infettive, Policlinico "Tor Vergata", Roma, Italy; 19 III Divisione di Malattie Infettive I.N.M.I “Lazzaro Spallanzani”, Roma, Italy; 20 IV Divisione di Malattie Infettive I.N,M.I “Lazzaro Spallanzani”, Roma, Italy; 21 Clinica delle Malattie Infettive, Dipartimento di Scienze della Salute, Polo Universitario San Paolo, Università degli Studi, Milano, Italy; University of New South Wales, Australia

## Abstract

**Background:**

Immunological non-responders (INRs) lacked CD4 increase despite HIV-viremia suppression on HAART and had an increased risk of disease progression. We assessed immune reconstitution profile upon intensification with maraviroc in INRs.

**Methods:**

We designed a multi-centric, randomized, parallel, open label, phase 4 superiority trial. We enrolled 97 patients on HAART with CD4+<200/µL and/or CD4+ recovery ≤25% and HIV-RNA<50 cp/mL. Patients were randomized 1:1 to HAART+maraviroc or continued HAART. CD4+ and CD8+ CD45+RA/RO, Ki67 expression and plasma IL-7 were quantified at W0, W12 and W48.

**Results:**

By W48 both groups displayed a CD4 increase without a significant inter-group difference. A statistically significant change in CD8 favored patients in arm HAART+maraviroc *versus* HAART at W12 (p=.009) and W48 (p=.025). The CD4>200/µL and CD4>200/µL + CD4 gain ≥25% end-points were not satisfied at W12 (p=.24 and p=.619) nor at W48 (p=.076 and p=.236). Patients continuing HAART displayed no major changes in parameters of T-cell homeostasis and activation. Maraviroc-receiving patients experienced a significant rise in circulating IL-7 by W48 (p=.01), and a trend in temporary reduction in activated HLA-DR+CD38+CD4+ by W12 (p=.06) that was not maintained at W48.

**Conclusions:**

Maraviroc intensification in INRs did not have a significant advantage in reconstituting CD4 T-cell pool, but did substantially expand CD8. It resulted in a low rate of treatment discontinuations.

**Trial Registration:**

ClinicalTrials.gov NCT00884858 http://clinicaltrials.gov/show/NCT00884858

## Introduction

The main goal of the highly active antiretroviral therapy (HAART) is the complete suppression of HIV replication and the increase of the CD4+ T cell count. 

Some observational studies demonstrated that at least 76% of patients initiating HAART achieved an undetectable viral load within 6 months [1], but a percentage of 9%-45% did not obtain an appropriate recovery of CD4+ T cells [2,3]. This situation, commonly referred to as immuno-virological discordance, mainly associated with a low CD4+ nadir, may lead to an increased risk of progression to AIDS defining illness and death [4-8]. 

It has been demonstrated that a lack of CD4 cells recovery and disease progression may be due to a persistent immune activation [9-12].

Several attempts of HAART intensification have been carried out to enhance the CD4 count recovery and the viral replication control. Some studies, like SILCAAT and ESPRIT, demonstrated that the use of interleukin IL-2 in association with antiretroviral therapy yielded no clinical benefit despite a substantial and sustained increase in the CD4+ cell count [13]. Abacavir, tenofovir, efavirenz and more recently raltegravir have been used as intensification drugs associated with HAART with no significant impact on the CD4+ cells rise [14-16]. 

One of the last antiretrovirals launched in the clinical arena was maraviroc (MVC), a CCR5 antagonist, that has been shown to have anti-inflammatory activity. The drug could have a potential role in the down-regulation of HIV-associated chronic inflammation by blocking the recirculation and trafficking of macrophages and monocyte-derived dendritic cells [17].

Few studies have been performed with MVC used as an intensification drug in patients with an insufficient immune response notwithstanding virological successes [18] and few observations could be done due to the small number of enrolled patients. Here we present a multi-centric randomised trial involving 97 immunological non responder (INR) patients, where MVC was administered in 47 patients as intensification treatment with the aim of increasing their CD4 count and eventually improving their immune competence.

## Materials and Methods

The protocol for this trial and supporting CONSORT checklist are available as supporting information; see Checklist S1 and Protocol S1.

Written informed consent was obtained from all participants. The study was performed in accordance with The International Conference on Harmonization Good Clinical Practice guidelines and applicable local regulatory requirements and laws.

### Study design

This was a multi-centric, randomized, parallel, open label, phase 4 superiority trial. The study was designed with a 48 week treatment period; enrollment started in April, 2009 and study completion was in April, 2011. One hundred and two HIV-1-infected adult patients were enrolled in 20 clinical centers coordinated by the Department of Biomedical and Clinical Sciences “Luigi Sacco”, Infectious Diseases Unit, University of Milan, Italy. Clinical trial identification n. NCT00884858 (registered on ClinicalTrials.gov).

At the screening visit, safety laboratory tests were conducted and previous antiretroviral treatment was assessed. Individual patients data and samples were collected and processed by each of the 20 participating clinical centers. At the time of randomization, eligible patients were randomly assigned in a 1:1 ratio to receive MVC for intensification of the current HAART regimen or HAART alone. The trial required a centre-stratified block-permuted randomization. The random allocation sequence was generated by the statistician. Study participants were enrolled by the physicians at the clinical centers and study participants were assigned to interventions by the coordinating center. MVC dose was decided according to the pharmaceutical company’s indications based on drug-drug interactions with other antiretrovirals. Plasma HIV-RNA was amplified with Amplicor HIV-1 Monitor Kit v1.5 and quantified by ultrasensitive real time PCR; this was performed at the Tor Vergata University I.D. research laboratory. 

Plasma and PBMCs samples were collected at screening, baseline, week 12, week 24 and week 48. Cell viability after thawing was assessed measuring 7AAD (Becton Dickinson) by flow cytometry (FC500 cytometer, Beckman Coulter). Only cells with viability >70% were used for the subsequent analyses. T-cells and PBMCs were drawn at the same time of day in all patients, in order to reduce the effect of diurnal variation. Tropism test was performed on PBMCs at screening on the available samples. For sequencing the HIV-1 gp120 V3 domain, HIV-1 DNA was extracted from 200 μL of PBMCs using a QIAamp DNA Minikit (Qiagen, Hilden, Germany), following the manufacturer's instructions. The HIV-1 *env* fragment including the V3 region was amplified by PCR using gp-120 primers and the Trugene core reagents kit (Siemens Healthcare Diagnostics Inc., Deerfield, IL, USA) [19]. Tropism testing was performed in single and FPR cut-off was 20%. This analysis was performed at the Luigi Sacco virology laboratory.

### Study population

All the enrolled patients were at least 18 years-old, HIV-1 infected adults with a CD4+ count <200/µL and/or CD4+ recovery ≤25% and HIV-RNA constantly <50 cp/mL who had been experiencing a HAART regimen for at least one year.

### Ethics statement

The central ethical approval, as communicated to the Italian Ministry of Health was provided by the Comitato Etico per la Sperimentazione Clinica (IRB) at “Luigi Sacco” Hospital, Milan, Italy. All the ethics committees/institutional review boards of the participating centres approved the study protocol. A complete list of the IRBs is available as a supporting information file.

### Efficacy analyses

The study’s primary endpoint involved the evaluation of the CD4+ cell count change from baseline to week 12 and week 48. In particular we established a simple endpoint that was the achievement of a CD4+ count > 200 cells/µL at two different measurements, and a composite endpoint including the achievement of a CD4 count> 200 cells/µL and a CD4+ cells recovery >25%. The endpoints were defined completely satisfied when HIV-RNA remained constantly <50 copies/mL over time.

### Flow cytometry

Lymphocyte surface phenotypes were evaluated by flow cytometry on frozen PBMCs (FC500; Beckman Coulter, Hialeah, FL), with the following fluorochrome-labelled antibodies: CD4-PE-Cy7, CD8-PE-Cy5, CD38-PE, HLA-DR-FITC, CD127-PE (Instrumentation Laboratory, Milan, Italy), CD45RA-FITC, CD62L-PE, Ki67-FITC, 7-AAD (Becton Dickinson Italia, Milan, Italy). We measured activation (HLA-DR/CD38) proliferation (Ki67), differentiation (CD127) and maturation (CD45RA/CD62L) on CD4+ and CD8+ T-cells. The following combinations were used: CD4/CD8/CD38/HLA-DR, CD8/CD4/Ki67, CD8/CD4/CD127 and CD8/CD4/CD45RA/CD62L. All immunological analyses were performed in the Laboratory at the Clinic of Infectious Diseases, University of Milano, San Paolo “Hospital”. The laboratory is fully equipped with all the reagents and supplies required for processing and studying PBMCs and plasma samples. 

### ELISA assays

Plasma levels of Interleukin (IL)-7 were quantified by commercially available ELISA (R&D System, Minneapolis, MN), according to manufacturer's instructions.

### Statistical analyses

We hypothesized 5% of “responder” patients in the control arm and δ = 20% in the immunological response between the 2 arms favoring the maraviroc arm (responder patients in the maraviroc arm = 25%) at week 48. For this superiority study we set the power as 1-β=80% with one-side α=0.05, thus 50 patients per arm were necessary, after considering protocol and screening violations and subjects lost to follow-up. Comparison of CD4+ and CD8+ count between the study arms A and B and at the different time-points were assessed by t-test for independent and dependent samples respectively in an intention to treat (ITT) analysis. Statistical evaluation of simple and composite endpoints was analyzed by binary logistic regression models for the odds ratio estimation and the related 95% confidence intervals. Odds ratios of the MVC arm was also controlled in the regression models for CD4+ counts at baseline, due to unbalancing though not statistically significant, of this parameter in the two study arms. Statistical significance level was estimated by Wald test and was set at .05 for all of the analyses. In the per-protocol (PP) analysis, patients who dropped the study or switched treatment were included until their last observation in the randomization arm. Baseline characteristics were collected and analyzed through chi-square, Fisher’s exact test and Mann Whitney U non-parametric test.

Immunological parameters at baseline (T0), after 12 (w12) and 48 weeks (w48) of MVC intensification were analyzed with GraphPad 5 PRISM software (version 5). All continuous variables were presented as median and interquartile ranges (25^th^-75^th^ percentile). The Mann Whitney U test was used for the comparison of immunological parameters (CD127, CD45RA, Ki67, HLA-DR, CD38 on CD4 and CD8 and plasma IL-7) between the 2 groups (ARM A *vs* ARM B). Wilcoxon signed-rank test was used to assess changes over time in the immunological parameters (CD127, CD45RA, Ki67, HLA-DR, CD38 on CD4 and CD8 and plasma IL-7) within the same study group (baseline *vs* W12 *vs* W48). All tests were 2-sided and differences were considered statistically significant at p<0.05. Bonferroni correction was applied to data of activation markers to account for test multiplicity. 

## Results

### Population characteristics and patients disposition

Of the 102 adult HIV-1 infected patients screened, 97 patients were enrolled in the study. 47 patients were randomized in arm A (HAART + MVC) and 50 in arm B (HAART). Recruitment took place from April, 2009 to April, 2010 and follow-up lasted for another 48 weeks. 

**Figure 1:**
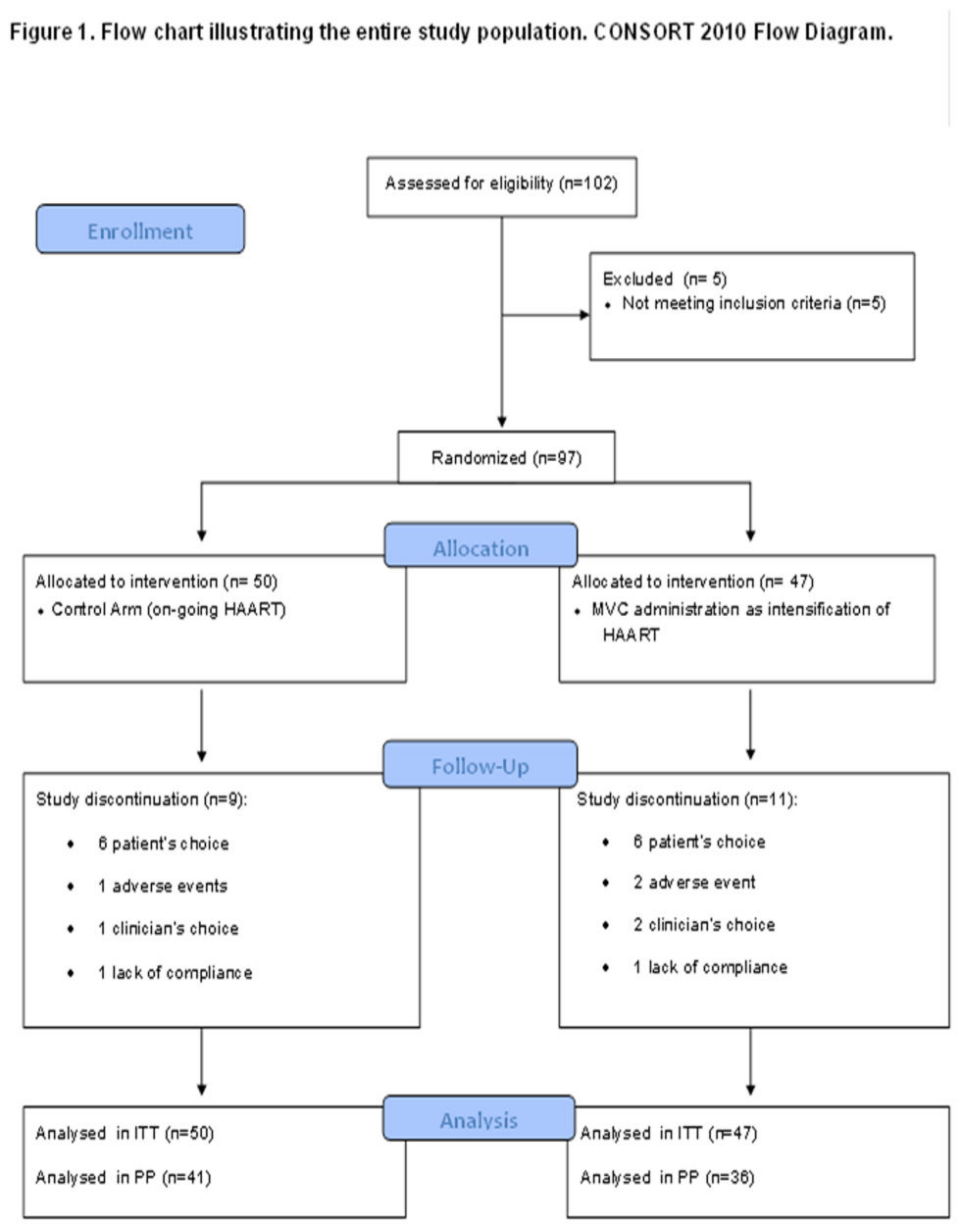
Flow chart illustrating the entire study population.

Twenty patients (11 in arm A and 9 in arm B) abandoned the study over time. Reasons for discontinuation were: patient’s choice (arm A: 6 patients vs arm B: 6 patients), adverse events (arm A: 2, arm B: 1) clinician’s choice (arm A: 2 vs arm B: 1) and lack of compliance (arm A: 1, arm B: 1) [Figure 1]. In particular, in arm A the side effects which caused the drop out from the study and the interruption of MVC in 2 patients where there was an onset of a Moskowitz-like syndrome in autoimmune thrombocytopenia [20]. and an allergic angioedema. The adverse event in arm B was diabetes mellitus type 2 and metabolic toxicity. Six patients changed their HAART during the 48 week follow up (physician’s choice, virologic failure, or adverse events): 5 in arm A and 1 in arm B. No clinical progression nor IRIS onset was observed. Out of the original 97 patients randomized, 90 (45 in each arm) had a complete dataset at 48 weeks and formed the final ITT analysis population. Baseline characteristics of the study population are summarized in Table 1. Thirty-six patients in arm A and forty-one patients in arm B (77 patients) were available for the PP analysis; in this analysis the median CD4 cell count at baseline was 191 cell/ µL in arm A and 170 cell/ µL in arm B and the median CD8 cell count at baseline was 566 cell/ µL in arm A and 731 cell/ µL in arm B. 

**Table 1:** Baseline characteristics at randomization in the ITT analysis population.

		CTRL	MVC	
		N=45	N=45	p
CDC	A/B	19 (42,2)	27 (60,0)	.140
	C	26 (57,8)	18 (40,0)	
Sex	Male	41 (91,1)	33 (73,3)	.051
	Female	4 (8,9)	12 (26,7)	
AZT	No	39 (86,7)	41 (91,1)	.502
	Yes	6 (13,3)	4 (8,9)	
Cotrimoxazole	No	26 (57,8)	21 (46,7)	.291
	Yes	19 (42,2)	24 (53,3)	
ddI+TDF	No	45 (100)	45 (100)	-
HCVAb	No	29 (64,4)	32 (71,1)	.499
	Yes	16 (35,6)	13 (28,9)	
HIV+duration	Years	11 (1-27)	12,5 (2-25)	.777
CD4 @ BL		170 (30-470)	191 (50-326)	.134
CD8 @ BL		731 (71-1.840)	566 (60-1.209)	.040
Nadir CD4		40 (5-193)	51 (1-160)	.709
VL	Log_10_	1,59 (1,28-1,84)	1,69 (1,28-1,69)	.436
VL<50 cp/mL duration	Months	36 (12-204)	37 (12-168)	.949
HAART duration	Months	63,5 (12-228)	103 (12-225)	.189
CD4 <200/μL duration	Months	33 (2-216)	40 (0-225)	.995
HAART regimen	NNRTI	11 (24,44 %)	14 (31,11%)	.480
	PI	28 (62,22%)	26 (57,78%)	.667
	PI+RAL	5 (11,11%)	2 (4,44%)	.238
	RAL	0 (0%)	2 (4,44%)	.153
	Others	1 (2,22%)	1 (2,22%)	.000

CTRL: controls; MVC: maraviroc; AZT: zidovudine; ddI: didanosine; TDF: tenofovir; BL:baseline; cp: copies; mL: milliliters; μL: microliters; HAART: highly active antiretroviral therapy; NRTI: Nucleoside Reverse Transcriptase Inhibitor; PI: protease inhibitor; NNRTI: Non-nucleoside Reverse Transcriptase Inhibitor; RAL: raltegravir; VL: HIV-RNA. Numbers are N (%) or median (min-max). Chi-square test was used for comparisons.

*= ***HAARTregimen***: NNRTI = Backbone + NNRTI; PI = Backbone + PI; RAL = Any HAART regimen containing RAL, with or without standard backbone, but without PI; Other = Only NRTIs-based regimens.

### T cell CD4+ and CD8+ count

**Figure 2:**
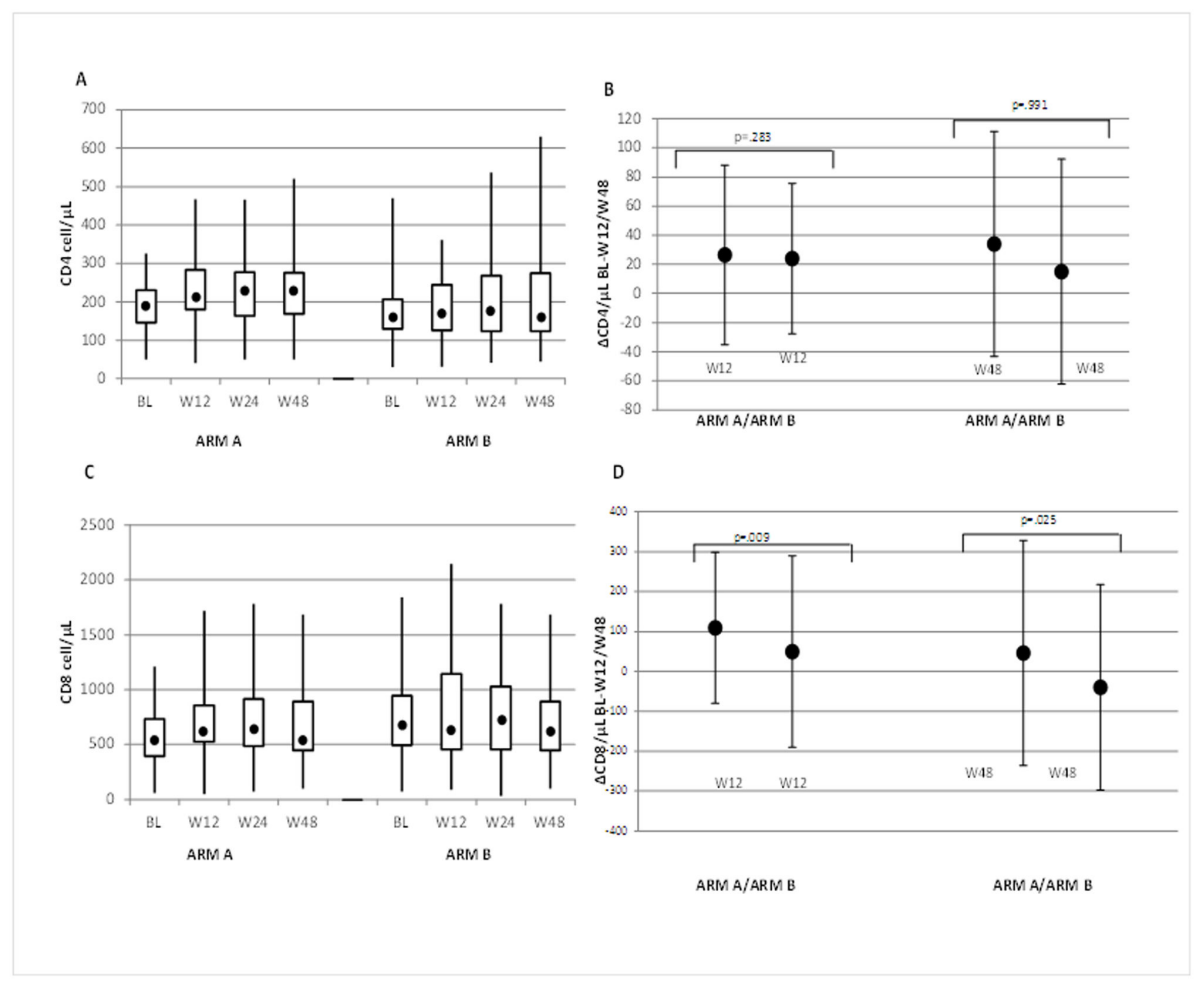
Plasma CD4+/CD8+ count and overtime change at baseline, week 12, and week 48 in the two study arms in the intention to treat analysis. **A** CD4+ count at baseline at week 12 and week 48 in arm A and B on the ITT analysis. CD4+ ITT analysis evidenced: in arm A a median count of 192 cell/µL at baseline, 212 cells/µL at week 12 and 223 cells/ µL at week 48; in arm B a median count of 169 cell/µL at baseline, 175 cells/µL at week 12 and 169 cells/µL at week 48. Box-plots (25% and 75% values) are presented. **B** CD4+ count change from baseline at week 12 and week 48 in the two study arms in the intention to treat analysis. The vertical lines represent the standard deviation. **C** CD8+ count at baseline at week 12 and week 48 in arm A and B on the ITT analysis. CD8+ ITT analysis evidenced: in arm A a median count of 580 cells/µL at baseline, 634 cells/µL at week 12 and 585 cells/µL at week 48; in arm B a median count of 725 cells/µL at baseline, 681 cells/µL at week 12 and 687 cells/µL at week 48. Box-plots (25% and 75% values) are presented. **D** CD8+ count change from baseline at week 12 and week 48 in the two study arms in the intention to treat analysis. The vertical lines represent the standard deviation.

CD4 counts used for the ITT analysis ( 90 patients) are shown in Figure 2A. In both arms a slight increase of the CD4+ count was registered (arm A: +26.5 cells/µL at week 12 and +34 cells/µL from baseline at week 48 and arm B: +24 cells/µL at week 12 and +15 cells/ µL at week 48) but no statistical significance was seen between the two arm at week 12 (p= 0.283) and week 48 (p=0.991) [Figure 2B]. Similar results were observed in the PP analysis (arm A: +26.5 cells/µL at week 12, +34 cells/µL at week 48, arm B:+20 cells/µL at week 12, +20 cells/µL at week 48, p= 0.200 between the two arms at week 12 and p= 0.637 at week 48).

CD8+ cell count was also evaluated at baseline, week 12 and week 48 in the ITT analysis [Figure 2C]. A significant increase of the CD8+ count was obtained in the intensification arm from baseline to week 12 as compared to the arm B (arm A: +109 cells/µL, arm B +49,5 cells/µL p= 0.009), this trend was confirmed at week 48 (arm A: +46 cells/µL, arm B: -40 cells/µL from baseline, p= 0.025) [Figure 2 D]. A same trend was observed in PP analysis at week 12 (arm A: +109 cells/µL, arm B: +49.5 cells/µL from baseline, p= 0.007) and week 48 (arm A: +46 cells/µL, arm B: -70 cells/µL from baseline, p= 0.003).

### HIV RNA quantification

In a population of 33 patients (18 in ARM A; 15 in ARM B) HIV RNA was quantified by ultrasensitive PCR, which was able to detect down to 1 copy/mL. In ARM A the median number of HIV-RNA copies/ml was 2 (IQR: <1-9.5) at T0; the same situation was observed in ARM B (median number copies/mL 2; IQR: <1-11). In ARM A the median number of HIV-RNA copies/mL was 3 (IQR: <1-10.5) at week 48, whereas it was <1 (IQR: <1-<1) in the arm B. A statistical significant reduction of HIV-RNA copies/mL was seen in arm B from T0 to W48 (p= .020), whereas no change was observed in arm A. At baseline, these viruses were: n. 14 CXCR4-tropic and n. 15 CCR5-tropic, without differences in median ultrasensitive HIV-RNA quantification.

### Efficacy of MVC intensification

At week 12 among 78 patients with available data a percentage of 64.3% of patients in arm A vs 38.9% of patients in arm B satisfied the simple endpoint without statistical significance (p=0.24, OR= 2.11, 95% CI from 0.61 to 7.31), 23.8% of patients in arm A vs. 25.0% of patients in arm B satisfied the composite endpoint without statistical significance (p=0.619, OR=0.76, 95% CI from 0.26 to 2.24). At week 48 a percentage of 62.2% of 45 patients in arm A vs 35.6% of 45 patients in arm B satisfied the simple endpoint without statistical significance (p=0.076, OR= 2.53, 95% CI from 0.91 to 7.07), 37.8% of patients in arm A vs 24.4% of patients in arm B satisfied the composite endpoint without statistical significance (p=0.236, OR=1.76, 95%CI 0.69-4.45) (Table 2). All ORs were adjusted for CD4+ cells at baseline.

**Table 2:** Immunological recovery as primary endpoint in the ITT analysis.

	At W 12						At W 48					
	CD4>200/µL			CD4>200/µL and recovery from baseline >25%			CD4>200/µL			CD4>200/µL and recovery from baseline >25%		
	p	OR	95%CI	p	OR	95%CI	p	OR	95%CI	p	OR	95%CI
ARM B ARM A	.24	2.11	.61-7.31	.619	0.76	.26-2.24	.076	2.53	.91-7.07	.236	1.76	.69-4.45

Differences in simple and composite endpoints between arm A and arm B were assessed at week 12 and week 48 in the intention-to-treat (ITT) analysis adjusted with the CD4 cell count at baseline. W: week; OR: odds ratio; CI: confidence interval; μL: microliters; ARM A: HAART + MVC; ARM B: HAART.

In the ITT analysis, both the simple and composite endpoints were satisfied by 15/55 (27.3%) of patients with CD4+ cells < 200 cell/µL at baseline. In these subgroup of patients the OR was 3.93 (p=0.048, 95%CI 1.01-15.28). In the PP analysis the scenario was similar to the ITT analysis with the exception of a statistical significant achievement of the simple endpoint at week 48 (OR=3.64; p=0.042, 95%CI 1.05-12.64) (Table 3).

**Table 3:** Immunological recovery as primary endpoint in the PP analysis.

	At W 12						At W 48					
	CD4>200/µL			CD4>200/µL and recovery from baseline >25%			CD4>200/µL			CD4>200/µL and recovery from baseline >25%		
	p	OR	95%CI	p	OR	95%CI	p	OR	95%CI	p	OR	95%CI
ARM B ARM A	.160	2.49	.70-8.91	.995	1.00	.32-3.11	.042	3.64	1.05-12.64	.120	2.36	.80-4.45

Differences in simple and composite endpoints between arm A and arm B were assessed at week 12 and week 48 in the per protocol (PP) analysis adjusted with the CD4 cell count at baseline. W: week; OR: odds ratio; CI: confidence interval; μL: microliters; ARM A: HAART + MVC; ARM B: HAART.

### Virological analysis between the study arms

Viral failure was defined as detection of HIV-RNA above the detection threshold confirmed by at least one other following measurement. It was observed in 3 patients: 2 in arm B and 1 in arm A. In particular, one patient in arm B showed detectable HIV-RNA values below 200 cp/mL from baseline to week 36 and the other one got a viral load of 1,130 cp/mL at week 12. The patient with viral failure in arm A at week 48 was censored in the PP analysis at this time point because of an adverse event which led to HAART interruption. 

Genotypic tropism test gave a positive result on PBMCs in 51 patients (arm A: 26, arm B: 25) at baseline: 25 were CCR5-tropic and 26 were CXCR4-tropic. Among all patients with a CCR5 tropism, the simple and composite endpoints were achieved with no statistical significance (p= 0.54, OR: 1.46 CI 0.44-4.91, p= 0.743, OR: 0.81 CI 0.22-2.93 respectively).Patients with CD4+ cell count less than 200 cells/µL at baseline with a CCR5 tropism got the simple and opposite endpoints with the same statistical significance (p= 0.760, OR: 1.30 [0.25-6.84]). The intensification with MVC corresponded to an increase in CD4 cell counts >200 cells/µL (simple end-point) when the entire cohort of 51 patients was considered (p= 0.023).

### Immunological analyses

These analyses were conducted in the entire ITT population.

#### T-cell immunephenotype

**Figure 3:**
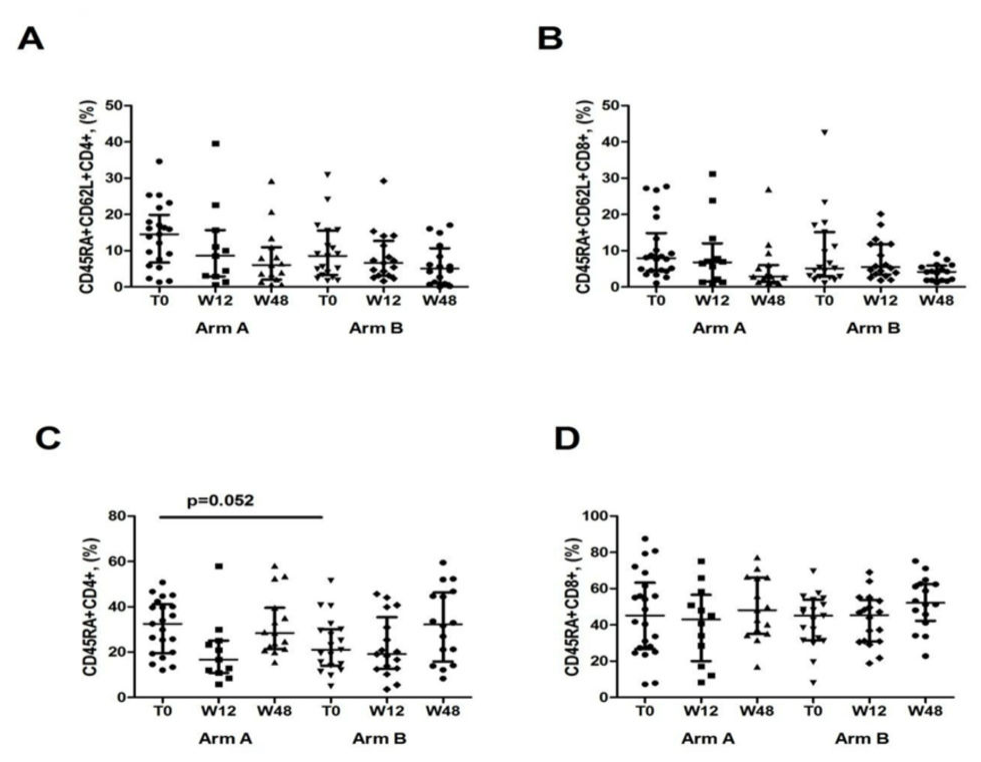
Naive and memory T-cell phenotypes in Immunological Non Responders with and without HAART intensification with MVC. **A**. Percentage of naive CD45RA+CD62L+CD4+ T-cells. **B**. Percentage of naïve CD45RA+CD62L+CD8+ T-cells. **C**. Percentage of memory CD45RA-CD4+ T-cells. **D**. Percentage of memory CD45RA-CD8+ T-cells. The vertical lines represent median and interquartile ranges (25^th^ and 75^th^ percentiles).

Between T0 and w12, both arm A and arm B displayed no changes in the proportion of naive CD45RA+CD62L+CD4+T-cells, with no substantial changes at w48. In particular, the percentage of naive CD45RA+CD62L+CD4+T-cells between T0 and W12 in arm A was: 14.50 [6.7-19.85]% vs 8.6 [2.9-15.7]%, p=.49; in arm B: 8.5 [3.3-15.5]% vs 6.6 [3.1-12.7]%, p=.91. At week 48 the percentage was in arm A, 6 [2-10.9]%, p=.64 and in arm B, 5.05 [1-10.65]%, p=.81. No changes over time in the proportion of CD4+ naive T-cells, expressing CD45RA and CD62L was shown in Arm B and Arm A (Figure 3A).

Similarly, no changes were seen in naïve CD45RA+CD62L+CD8+ in the two study groups at w12 and w48. In arm A at T0, 7.9 [4.5-14.8]% vs at w12, 6.7 [1.5-12]%, p=.46; in arm B: T0, 5.1 [2.8-15.1]% vs w12, 5.5 [3.4-11.8]%, p=.85 and at w48 in arm A: 2.9 [1.4-6]%, p=.25 and in arm B: 4.1 [1.8-5.8]%, p=.47 (Figure 3B).

As for CD45RA-CD4+ memory T-subset, arm A and arm B patients displayed no changes over time: arm A: T0, 67.4 [59.8-80.4]%, w12, 83.3 [74.9-89.2]%, w48, 71.6 [60.4-78.8] p=.08 for T0 vs w12, p=1 for T0 vs w48. Arm B: T0, 78.9 [69.8-85.9]%, w12, 80.9 [64.5-87.3]%, w48, 67.80 [53.7-84.1]%, p=.97 for T0 vs w12, p=.37 for T0 vs w48. At T0, arm A seemed to have higher levels of CD45RA-CD4+ as compared to arm B (p=.052), no other inter-group differences at w12 and w48 were shown (p=.65; p=.91, respectively (Figure 3C).

No changes in the proportion of CD45RA-CD8+ were observed between T0 and w12 in arm A (48.75 [36.7-72.8]% vs 57.1 [43.5-79.9]%, p=.12) and arm B (57.1 [46.1-68.5]% vs 54.7 [48.9-69.1]%, p=.68. A similar trend was seen at w48 (arm A: 51.9 [33.9-65.8]%, p=.74; arm B: 47.80 [37.5-57.8]%, p=.22 (Figure 3D).

#### T-cells activation and proliferation

**Figure 4:**
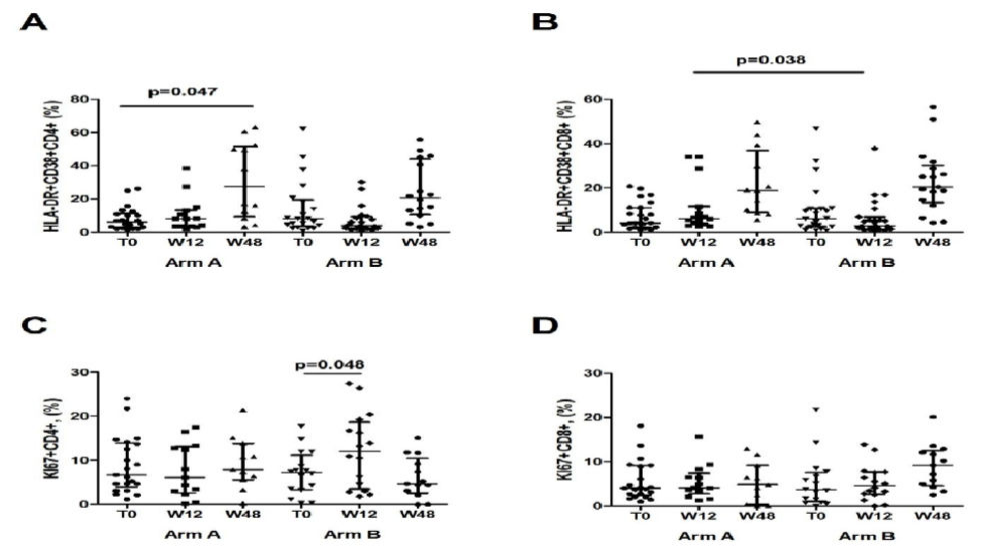
T-cells activation and proliferation in Immunological Non Responders with and without HAART intensification with MVC. **A**-**B**. T-cell activation defined as the co-expression of HLA-DR and CD38 on CD4+ and CD8+. **C**-**D**. T-cell proliferation defined as the expression of Ki67 on CD4+ and CD8+. The vertical lines represent median and interquartile ranges (25^th^ and 75^th^ percentiles).

Arm A displayed an increased proportion of activated HLA-DR+CD38+CD4+ from a median of 6.1 (2.9-11.33)% at baseline to 8.2 (3.7-13.5)% at w12 to 27.4 (9.3-51.7)% at w48, reaching significance only at w48 (p=.47, p=.047; respectively, whereas no changes in HLA-DR+CD38+CD4+ were shown in Arm B (T0, 8.1 [3.6-19.5] %; w12, 4 [2.8-9.4]%; w48, 20.6 [10.7-44.2]%; p=.29 for T0 vs w12; p=.17 for T0 vs w48 (Figure 4A). No changes in the proportion of activated HLA-DR+CD38+CD8+ between T0, w12 and w48 was seen in arm A (T0, 4.2 [2.3-11]%; w12 5.9 [3.5-11.5]%; w48 18.9 [8.9-36.9]% p=.64 for T0 vs w12; p=.11 for T0 vs w48) and in arm B (T0,6.2 [2.5-10.7]%; w12 2.8 [1.5-6.9]%; w48 20.4 [13.3-30.2]%, p=.13 for T0 vs w12; p=.07 for T0 vs w48). At T12, arm A showed higher levels of HLA-DR+CD38+CD8+ as compared to arm B (p=.038); no other inter-group differences were shown at T0 and w48 (p=.95; p=.79; respectively) (Figure 4B).

Between T0 and w12, no changes in Ki67+CD4+ were observed in arm A (6.7 [3.9-13.9]% vs 6.1 [2.6-13.1]%, p=.42), whereas arm B displayed an increase in Ki67+CD4+ (7.2 [3.3-11.2[% vs 12.1 [3.5-18.7]%;p=.048). No changes between T0 and w48 in the two study groups were seen (arm A: 7.9 [5.5-13.8]%, p=.22 ; arm B: 4.7 [2.6-10.5]% p=.99) (Figure 4C). 

With regard to Ki67+CD8+ no changes were seen in the two study groups at w12 (arm A: 4 [2.4-9.1]% vs 4.1 [2.8-7.4]%, p=.45; arm B: 3.7 [0.9-7.5]% vs 4.6 [2.6-7.7]%, p=.69) and w48 (arm A: 2.9 [1.4-6]%, p=.81; arm B: 4.1 [1.8-5.8]%, p=.16) (Figure 4D).

#### IL-7/IL-7R system

**Figure 5:**
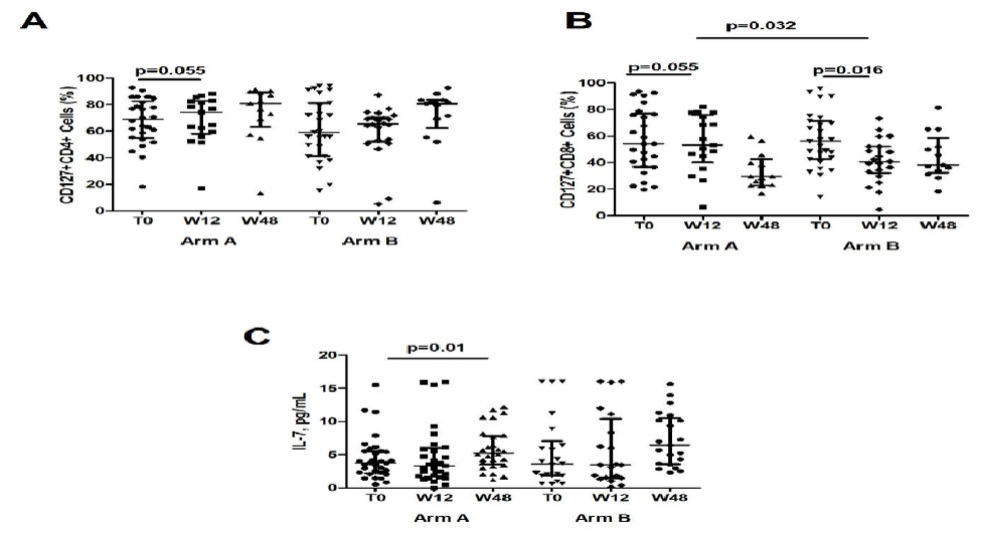
IL-7/IL-7R system in Immunological Non Responders with and without HAART intensification with MVC. **A**. Percentage of CD127+CD4+ T-cells. **B**. Percentage of CD127+CD8+ T-cells. **C**. Plasma levels of IL-7. The vertical lines represent median and interquartile ranges (25^th^ and 75^th^ percentiles).

Arm A displayed an increasing trend in CD127+CD4+ T-cells, close to statistical significance at w12 (T0, 69 [54.9-82.3]%, w12, 74.2 [58.1-82.8]%, w48 80.9 [63.2-89.3]%; p=.055 for T0 vs w12; p=.46 for T0 vs w48), whereas no changes was shown over time in arm B (T0, 59.2 [41.2-81.1], w12, 65.5 [52.4-70.4]%, w48, 80.7 [62.3-83.4]%, p=.39 for T0 vs w12, p=.16 forT0 vs w48) (Figure 5A).

Between T0 and w12, both arm A and arm B reduced the proportion of CD127+CD8+ (Arm A: 54.2 [36.9-76.9]% vs 53.2 [40.1-75.9]%; p=.055; Arm B: 56.1 [42.9-71.5]% vs 40.3 [32.3-52.2]%; p=.016), with arm A displaying higher CD127+CD8+ T-cells as compared to arm B at w12 (p=.034), despite similar baseline levels (p=.96). At w48, the CD127+CD8+ reduction was no longer significant in arm A (29.5 [22.8-42.6]%; p=.31) as well as in arm B (38.1 [32.5-58.5]%; p=.07), with arm A still maintaining a trend to higher CD127+CD8+ (p=.08) (Figure 5B).

Interestingly, while arm A displayed a significant increase of circulating IL-7 at w48 (T0, 3.3 [2.2-15.5] pg/mL vs w12 3.3 [1.7-6] pg/mL; p=.47;vs w48 7.8 [5.2-12.2] pg/mL; p=.01 no changes were observed in arm B(T0, 3.6 [1.8-7.04] pg/mL vs w12, 3.4 [1.5-10.4] pg/mL; p=.94; vs w48 6.43 [3.6-10.5] pg/mL; p=.23 (Figure 5C).

## Discussion

Our study hypothesized a possible role of MVC in enhancing an immunological recovery in HIV-1 infected patients with a stable viral load suppression on HAART but with a poor CD4+ count recovery after several years of antiretroviral treatment.

In our study, we observed that HAART intensification with MVC didn’t lead to a significant increase of the CD4+ count. In particular we didn’t observe any particular differences between arm A and B at week 12 and 48 with the exception of a slight increase of the CD4+ cells count in arm A at week 12 which was not confirmed at week 48. A CD4+ T-cells recovery at the early stage of MVC intensification was reported in other studies.

Massanella et al [21] demonstrated that naïve HIV- infected patients got a significant CD4+ count gain at week 12 when HAART was intensified with MVC. This trend was not confirmed at week 48. Cossarini et al [22] demonstrated a significant increase of CD4+ cells in multi-experienced patients who intensified HAART with MVC versus those on HAART +darunavir/ritonavir. 

A temporary increase of the CD4+ cells during the first period of MVC intake could be explained by a possible reduction of activated CCR5+ T-cells as a result of the CCR5 co-receptor blockage associated with viral suppression. This situation can promote a temporary CD4+ cell release from the lymphoid reservoir to plasma by a type of “redistribution” phenomenon. Another explanation could be that MVC blockage of the co-receptor can lead to a longer survival of CD4+ cells by modulating the apoptotic process or some other unknown mechanism as shown in a study lead by Wilkin et al. [23] 

HIV-RNA ultrasensitive quantification didn’t seem to be influenced by MVC intake over-time. A significant decrease of HIV-RNA was seen at W48 in ARM B; although the sample of patients was limited compared to the entire study cohort. This data may be explained by improving adherence within the control group after the enrollment period of the study. 

Some interesting results among the immunological markers should be cautiously interpreted due to inflation of type I errors caused by multiplicity. 

We didn’t find any change of naïve (CD45RA+) and memory (CD45RA-) CD4+ cells. Differently an increase of activation markers expression on CD4+ T-cells, HLA-DR and CD38, was seen at week 12 and better at week 48 (p=0.047). This finding confirmed what Hunt et al [24] had already demonstrated. In their study a population of “immunological non-responders” that intensified a HAART regimen with MVC showed an increase of the cellular activation both in the CD4+ and CD8+ cells of peripheral blood and more significantly in gut mucosa samples.

They also observed an increased expression of CCR5 on CD4+ and CD8+ cells suggesting that compensatory increases in CCR5 ligands might activate macrophages and that T cells through alternative chemokine receptors should be further explored. Hunt and colleagues studied the effect of MVC on MIP1β and evidenced an increase in its level over-time which returned to baseline upon MVC cessation [24].

We found a significant increase of CD8÷ cells in the intensification arm at week 12(p= 0.009) and week 48 (p=0.025). Immunological analyses performed on CD8 didn’t show any particular change in the naïve and memory CD8+ cells. The analysis of cellular activation of these cells through evaluation of HLA-DR and CD38 showed an increased expression of these markers in arm A at week 12 but not at week 48. Our observation was concordant with what Hunt et al had described. A more powerful action of MVC on CD8+ cells can be justified by a higher presence of CCR5 coreceptor on CD8+ than on CD4+ cells [25]. Blockage of CD8+ cells migration from peripheral blood to the tissues would allow their pool expansion. This had been observed in our study at week 12 and week 48. This conclusion has not been supported by other studies where a reduction of CD8+cells activation and loss of monocytes were seen in a population using MVC as intensification [26] or as a component of HAART [27]. A further consideration on the increase in CD8+ lymphocytes is that their redistribution could indeed represent an improvement of the immune system without mirroring an increased viral production. We did not observe any change of the proliferation marker Ki67+ at week 48, although a significant increase of this marker was unexpectedly seen at week 12 in arm B (p=.048). One limitation of the results for this parameter was the small size of the samples available for T-cell proliferation measurements; further analyses should be performed to confirm these preliminary data.

An increase of IL-7 level in arm A could suggest an effect of MVC in maintaining T-lymphocyte homeostasis through T-lymphocyte production in a milieu of stable turnover of peripheral T-cells, as evidenced by the absence of Ki67+ change over time, although Bonferroni analysis did not confirm a significant difference between the 2 arms. Whether or not raised IL-7 translates into increased *de novo* T-cell production [28,29] is not clear from our data, as we didn’t evaluate thymic output.

We also found an increased expression of IL-7 receptor on CD4+ cells in MVC-receiving patients; the finding of an inverse trend on CD8+ cells suggest diverse IL-7R regulation on circulating CD4+ and CD8+ upon MVC administration. This different behavior of IL-7R on CD8+ may be caused by a possible down-regulation of cell-surface receptor expression in the presence of raised levels of plasma IL-7. Further studies are warranted to better unravel the diverse regulation of IL-7/IL-7R axis upon MVC administration and by investigating the effect of MVC on IL-7R expression inducers and inhibitors [30].

MVC intensification of HAART did not show a significant efficacy in satisfying both the simple and composite endpoints with the intention to treat analysis adjusted with the CD4+ cells at baseline, although a trend towards the achievement of the simple endpoint was seen at week 48 in the on treatment analysis (p=0.076). The estimates of odds ratios resulted quite identical also when applying LOCF methods in the ITT analysis (data not shown). Of note, the subjects in the control arm achieved a CD4+ response higher than expected when the study was designed. Nevertheless, an interesting observation came up when we stratified the analysis according to patients with CD4+ cell count less than 200 cells/µL at baseline: we found the statistical significant achievement of simple and composite endpoints (p=0.048, OR 3.93, 95% confidence interval from 1.01 to 15.28).

Other studies tried to evaluate the immune system response to a HAART intensification with MVC in immunological non-responder patients [18,23,31]. All of these studies did not have a control arm, exhibited discordant results in increasing CD4+ cell counts, and had an observation period shorter than our study.

In our study, MVC was discontinued in 2/47 subjects and demonstrated a satisfactory control of viral replication: virological failures were seen equally in arms A and B.

There are several limitations to this study. This was a randomized trial which did not imply a placebo drug intake in the control arm. Changes in the immune response in arm A could have been influenced by a sort of “placebo effect” due to the consciousness of taking a forth drug in the regimen. Immunophenotyping analyses were performed on cryo-preserved PBMCs: the freezing process may have damaged the expression of cell surface proteins. Data on CD8 subsets would be interesting; unfortunately central *vs* effector memory CD8 phenotyping could not be addressed because there was no remaining biological material at the time when the original samples were taken. A number of confounding factors, such as different sample processing, shipping conditions, samples storage and lab blood tests data collected from several centers could have interfered with the quality of results. Moreover, 20% of our patients discontinued the study for several reasons and this could have impaired the final results of the protocol.

Although CD4+ T-cell counts improved in the MVC intensification arm, the primary end-points were not satisfied in this population of immunological non responders. MVC showed to be efficient in increasing the CD8+ cell count; this might mean a better recovery of the cell-mediated immunity and therefore a reduction of AIDS and non-AIDS related events. Further analyses are needed to confirm this last hypothesis.

## Supporting Information

Checklist S1 **CONSORT checklist.**
(DOC)

Protocol S1 **Protocol in English.**
(DOC)

Protocol S2 **Protocol in Italian.**
(DOC)

Text S1 **EC/IRBs.**
(DOC)
