# Supplementary material for: Maraviroc as Intensification Strategy in HIV-1 Positive Patients with Deficient Immunological Response: an Italian Randomized Clinical Trial
Source: PLoS One. 2013 Nov 14;8(11):e80157. doi: 10.1371/journal.pone.0080157 (PMC3828227; doi:10.1371/journal.pone.0080157)
Supplement: Checklist S1 — CONSORT checklist. (DOC) [file pone.0080157.s001.doc]

| Item No | **CONSORT 2010 checklist of information to include when reporting a randomised trial***  Checklist item | | | | Section/Topic | |  | |
| --- | --- | --- | --- | --- | --- | --- | --- | --- |
| 1a | Identification as a randomised trial in the title | | | | | Title and abstract |  |  |
| 1b | Structured summary of trial design, methods, results, and conclusions (for specific guidance see CONSORT for abstracts [1, 2]) | | | | |  |  |  |
| 2a | Scientific background and explanation of rationale | | | | | **Introduction** |  |  |
| 2b | Specific objectives or hypotheses | | | | | Background and objectives |  |  |
| 3a | Description of trial design (such as parallel, factorial) including allocation ratio | | | | | **Methods**  Trial design |  |  |
| 3b | Important changes to methods after trial commencement (such as eligibility criteria), with reasons | | | | |  |  |  |
| 4a | Eligibility criteria for participants | | | | | Participants |  |  |
| 4b | Settings and locations where the data were collected | | | | |  |  |  |
| 5 | The interventions for each group with sufficient details to allow replication, including how and when they were actually administered | | | | | Interventions |  |  |
| 6a | Completely defined pre-specified primary and secondary outcome measures, including how and when they were assessed | | | | | Outcomes |  |  |
| 6b | Any changes to trial outcomes after the trial commenced, with reasons | | | | |  |  |  |
| 7a | How sample size was determined | | | | | Sample size |  |  |
| 7b | When applicable, explanation of any interim analyses and stopping guidelines | | | | |  |  |  |
|  |  | | | | | Randomisation: |  |  |
| 8a | Method used to generate the random allocation sequence | | | | | Sequence generation |  |  |
| 8b | Type of randomisation; details of any restriction (such as blocking and block size) | | | | |  |  |  |
| 9 | Mechanism used to implement the random allocation sequence (such as sequentially numbered containers), describing any steps taken to conceal the sequence until interventions were assigned | | | | | Allocation concealment mechanism |  |  |
| 10 | Who generated the random allocation sequence, who enrolled participants, and who assigned participants to interventions | | | | | Implementation |  |  |
| 11a | If done, who was blinded after assignment to interventions (for example, participants, care providers, those assessing outcomes) and how | | | | | Blinding |  |  |
| 11b | If relevant, description of the similarity of interventions | | | | |  |  |  |
| 12a | Statistical methods used to compare groups for primary and secondary outcomes | | | | | Statistical methods |  |  |
| 12b | Methods for additional analyses, such as subgroup analyses and adjusted analyses | | | | |  |  |  |
| 13a | For each group, the numbers of participants who were randomly assigned, received intended treatment, and were analysed for the primary outcome | | | | | **Results**  Particpants flow (a diagram is strongly recommended) |  |  |
| 13b | For each group, losses and exclusions after randomisation, together with reasons | | | | |  |  |  |
| 14a | Dates defining the periods of recruitment and follow-up | | | | | Recruitment |  |  |
| 14b | Why the trial ended or was stopped | | | | |  |  |  |
| 15 | A table showing baseline demographic and clinical characteristics for each group | | | | | Baseline data |  |  |
| 16 | For each group, number of participants (denominator) included in each analysis and whether the analysis was by original assigned groups | | | | | Numbers analysed |  |  |
| 17a | For each primary and secondary outcome, results for each group, and the estimated effect size and its precision (such as 95% confidence interval) | | | | | Outcomes and estimation |  |  |
| 17b | For binary outcomes, presentation of both absolute and relative effect sizes is recommended | | | | |  |  |  |
| 18 | Results of any other analyses performed, including subgroup analyses and adjusted analyses, distinguishing pre-specified from exploratory | | | | | Ancillary analyses |  |  |
| 19 | All important harms or unintended effects in each group (for specific guidance see CONSORT for harms^28^) | | | | | Harms |  |  |
| 20 | Trial limitations, addressing sources of potential bias, imprecision, and, if relevant, multiplicity of analyses | | | | | **Discussion**  Limitations |  |  |
| 21 | Generalisability (external validity, applicability) of the trial findings | | | | | Generalisability |  |  |
| 22 | Interpretation consistent with results, balancing benefits and harms, and considering other relevant evidence | | | | | Interpretation  **Other information** |  |  |
|  | |  |  |  | | | | |
| 23 | Registration number and name of trial registry | | | | | Registration |  |  |
| 24 | Where the full trial protocol can be accessed, if available | | | | | Protocol |  |  |
| 25 | Sources of funding and other support (such as supply of drugs), role of funders **see submission system** | | | | | Funding |  |  |
|  |  | | | | |  |  |  |

*We strongly recommend reading this statement in conjunction with the CONSORT 2010 Explanation and Elaboration [3] for important clarifications on all the items. If relevant, we also recommend reading CONSORT extensions for cluster randomised trials, [4] non-inferiority and equivalence trials [5], non-pharmacological treatments [6], herbal interventions [7], and pragmatic trials [8]. Additional extensions are forthcoming: for those and for up to date references relevant to this checklist, see [www.consort-statement.org](http://www.consort-statement.org/).

References

1. Hopewell S, Clarke M, Moher D, Wager E, Middleton P, Altman DG, et al. CONSORT for reporting randomised trials in journal and conference abstracts. *Lancet* 2008;371:281-3.
2. Hopewell S, Clarke M, Moher D, Wager E, Middleton P, Altman DG, et al. CONSORT for reporting randomized controlled trials in journal and conference abstracts: explanation and elaboration. *PLoS Med* 2008;5:e20.
3. Moher D, Hopewell S, Schulz KF, Montori V, Gøtzsche PC, Devereaux PJ, et al. CONSORT 2010 Explanation and Elaboration: updated guidelines for reporting parallel group randomised trials. *BMJ* 2010;340:c869.
4. Campbell MK, Elbourne DR, Altman DG. CONSORT statement: extension to cluster randomised trials. *BMJ* 2004;328:702-8.
5. Piaggio G, Elbourne DR, Altman DG, Pocock SJ, Evans SJ. Reporting of noninferiority and equivalence randomized trials: an extension of the CONSORT statement. *JAMA* 2006;295:1152-60.
6. Boutron I, Moher D, Altman DG, Schulz KF, Ravaud P. Extending the CONSORT statement to randomized trials of nonpharmacologic treatment: explanation and elaboration. *Ann Intern Med* 2008;148:295-309.
7. Gagnier JJ, Boon H, Rochon P, Moher D, Barnes J, Bombardier C. Reporting randomized, controlled trials of herbal interventions: an elaborated CONSORT statement. *Ann Intern Med* 2006;144:364-7.
8. Zwarenstein M, Treweek S, Gagnier JJ, Altman DG, Tunis S, Haynes B, et al. Improving the reporting of pragmatic trials: an extension of the CONSORT statement. *BMJ* 2008;337:a2390

Legend. CONSORT: CONsolidated Standards of Reporting Trials.
