# Supplementary material for: Maraviroc as Intensification Strategy in HIV-1 Positive Patients with Deficient Immunological Response: an Italian Randomized Clinical Trial
Source: PLoS One. 2013 Nov 14;8(11):e80157. doi: 10.1371/journal.pone.0080157 (PMC3828227; doi:10.1371/journal.pone.0080157)
Supplement: Protocol S1 — Protocol in English. (DOC) [file pone.0080157.s002.doc]

# Study Protocol

## Title

## Use of Maraviroc (MVC) in immunogical non-responder HIV-1-infected patients

## Rationale

Suboptimal improvement in CD4 cell count is not uncommon in HIV-1-infected patients with suppressed plasma HIV-RNA levels, and a decrease in CD4 cell count in patients with suppressed or low level viremia has been observed.

Although the efficacy of current antiretroviral medications is well established, some antiviral combinations are very effective in suppressing HIV-1 load whereas do not exert any effect on immune reconstitution.

Both T-cell immune activation and fibrosis of peripheral lymphoid tissue could create an environment in which CD4 T cell count decrease in the setting of low or suppressed plasma viremia is likely to occur.

Another fascinating hypothesis, which has still to be elucidated, is that reconstitution of the depleted CD4 pool is blocked by an excess of gp120 HIV-1 protein. This extra-production could be counteracted by an inhibitor of the CCR5 co-receptor that represents one of the major docking tools of HIV-1.

From a clinical point of view, a post-hoc analysis of MERIT study, which compared safety and efficacy of Maraviroc versus Efavirenz in patients who were naïve for antiretroviral therapy, has shown a greater and faster increase of CD4 cells count in patient which were taking Maraviroc than in patients in Efavirenz treatment arm, and this data were statistically significant. Moreover, a larger proportion of patient with less than 200 CD4 cells/µL at baseline has reached or exceeded this threshold in the Maraviroc arm.

In patients who did not reach a virological suppression a minor probability to have AIDS-defining events and an increase of a CD4 cells count have been observed [2].

The increase of CD4 cells count in patients treated with Maraviroc *versus* placebo has been observed also with a different dosage of Maraviroc [3, 4] and in patients with Dual/Mixed or X4-tropic viral strains, in spite of not reaching a virological suppression [5, 6].

However, these observations have been conducted within studies with other primary objectives, while a study with the aim to directly verify this hypothesis has never been performed.

Similar observations have been done in patients who took Enfuvirtide [7], but a possible larger use of this drug has been probably limited by a poor tolerability, particularly due to subcutaneous administration, which causes frequent skin reactions in the injection site. We have chosen Maraviroc because of its ease of use, which makes it more suitable for an intensification strategy in a regimen which had already determined a virological success.

With this in mind, we would like to propose and design a pilot exploratory clinical trial involving a population of HIV-1-infected patients that rapidly reached a virologic suppression without a reconstitution of their immune system.

The chosen dosage of Maraviroc is standard for HIV-infection treatment, after considering necessary modifications according to concomitant medications, as specified in the product data sheet. There are also more data in terms of efficacy and safety for these dosages (from 150mg BID to 600mg BID). The one-year duration of our study is based on the fact that the CD4 cells count increase has been observed in previously reported clinical trials at 48 weeks of treatment. According to this observation, the selected duration would be sufficient to obtain the desired effect.

## Objectives

Primary objectives:

- Evaluate the **clinical (defined as protection from the onset of opportunistic infections) and immunological efficacy** of HAART intensification with MVC as treatment of HIV-1 infection in patients with a CD4 count ≤ 200 cells/μL and/or a recovery of CD4 cells < 25% compared to the HAART initiation and with a complete and stable virologic suppression after 12 months of HAART. Patients could also being included if their CD4 slope has been stable without any improvement, with an absolute value around 200 cells/μL;
- Evaluate the effects of HAART intensification with MVC on the modification of **immunologic and virologic parameters**;
- Evaluate the **tolerability** of HAART intensification with MVC and the appearance of drug-related side effects.

Secondary objectives:

- Evaluation of intensification treatment of HAART with MVC on modification of following immunological parameters: chemokines-mRNA analysis, helper memory and naïve CD4 lymphocytes, IL-7/IL-7R homeostatic system.

Endpoints

- Simple endpoint: achievement of CD4 count > 200/µL in two consecutive measurements;
- Composite endpoint: CD4 counts > 200/µL and recovery of CD4 > 25% at 2 consecutive time-points (w12 and w48).

## Design

This will be a randomised, multicenter, study that will evaluate HAART intensification with MVC as treatment of HIV-1 infection in patients with a CD4 count ≤ 200 cells/μL and/or a recovery of CD4 cells < 25% compared to the HAART initiation and/or a stable CD4 slope without any improvement, with an absolute value around 200 cells/μL and with a complete and stable virologic suppression after 12 months of HAART. Among the adult HIV-1 infected patients screened, 50 patients will be randomized in arm A (HAART + MVC) and 50 in arm B (HAART without MVC), in a 1:1 fashion.

Treatment regimen

Maraviroc dosing schedule is: from 150mg to 600mg BID, according to the other antiretroviral drugs in the patient.s therapy. Maraviroc will be administered at the dosing regimen of 150mg BID with nelfinavir and protease inhibitors boosted by ritonavir (PI/r), as example atazanavir/rtv, darunavir/rtv, lopinavir/rtv e saquinavir/rtv; 300mg BID with nevirapine, fosamprenavir/rtv, tipranavir/rtv, raltegravir and enfuvirtide (T-20); 600mg BID with efavirenz and etravirine. In the case of administration within a regimen including a PI/rtv (atazanavir/rtv, darunavir/rtv, lopinavir/rtv e saquinavir/rtv), MVC dosage will be always 150mg BID.

Study population

The enrollment of 100 continuously observed patients, with the characteristics mentioned above, at Clinical Science Department, Division of Infectious Diseases and Immunopathology of Università degli Studi, “L. Sacco” Hospital, Milan, is expected after EC/IRB approval.

Other clinical centers will participate to the study: Clinic of Infectious Diseases, “San Paolo” Hospital: Prof.ssa Antonella D’Arminio Monforte; 1^a^ and 2^a^ Division of Infectious Diseases, “L. Sacco” Hospital, Milan: Dr. Giuliano Rizzardini; Clinic of Infectious Diseases, Scientific Institute “San Raffaele” Hospital, Milan: Prof. Adriano Lazzarin; Clinic of Infectious Diseases, University of Brescia: Prof. Giampiero Carosi; Clinic of Infectious Diseases, University Tor Vergata, Rome: Prof. Massimo Andreoni; Clinic of Infectious Diseases, University of Turin: Prof. Gianni Di Perri; National Institute of Infectious Diseases, “L. Spallanzani” Hospital, Rome: Dr. Andrea Antinori; Clinic of Infectious Diseases, “San Gerardo” Hospital, Monza: Dr. Andrea Gori.

The enrollment will include 100 HIV-positive patients, with CD4+ lymphocyte count ≤200 cells/µL and/or CD4+ cells count recovery ≤25%, with suppressed viraemia (HIV-RNA <50 cp/mL) after 12 months of antiretroviral drugs. It will be possible also to enroll patient with stable CD4+ count without any improvement, with an absolute value around 200 cells/ µL.

## Timelines

The clinical, immuno-virological evaluation will be conducted at baseline, then after 1, 4, 8, 12 weeks from the enrolment, thereafter 4, 6 and 9 months from the enrolment, and at the end of the administration period.

Duration of study

The enrollment period will last for 6 months, and MVC administration will continue for 12 months.

Inclusion criteria:

- Male or female subjects <18 years old;
- Serological positivity for HIV infection determined by ELISA test and confirmed by Western Blot;
- CD4+ T cells lymphocyte count ≤200/µL and/or a recovery of CD4 cells < 25% compared to the HAART initiation and/or a stable CD4 slope without any improvement, with an absolute value around 200 cells/μL;
- Complete and stable virologic suppression (HIV-RNA <50 cp/mL) after at least 12 months of HAART;
- Negative pregnancy test at least 14 days before start of treatment. All patients will have to guarantee effective contraception methods in the period including 4 weeks before and 8 weeks after the treatment;
- Comprehension and underwriting of written informed consent.

Exclusion criteria:

- Known allergy/intolerance for any study drug;
- Any treatment with immunomodulatory drugs or with growths factors or cytokines (IL-2, α-, β-, γ-IFN, G-CSF, GM-CSF), made within a year from the potential screening;
- Current cancer or opportunistic infections;
- Cardiovascular disease or electrocardiographic abnormalities;
- Respiratory diseases, as asthma, COPD and chronic restrictive broncopneumopathy;
- Systemic treatment with steroid drugs within 4 weeks before starting the study treatment;
- Suspected or established chronic inflammatory or autoimmune disease;
- Active drug use, alcohol abuse or any patient who is considered poorly cooperative by the investigator;
- Detection of following laboratory parameters in the 2 weeks before starting the study treatment:
  - SGOT e SGPT ≥2,5 times over the superior reference limit;
  - Serum creatinine >1,5 times over the superior reference limit;
  - ANC <1000/μL;
  - Hemoglobin <10 g/dL;
  - Platelets <75.000/μL;
  - Reticulocytes >2%;
  - Karnofsky Index <50.

**Study Methodology**

Clinical evaluation.

At baseline, at weeks 2, 4, 8, 12 from the beginning of the study, following 4, 6 and 9 months from the beginning of the study and at the end of the period of administration of MVC:

- Clinical History;
- Physical Examination;
- Evaluation of Karnofsky index

Blood tests

At enrollment and subsequently at weeks 2, 4, 8, 12 from the beginning of the study, and then at 4, 6 and 9 months from the beginning of the study and at the end of the period of administration of MVC:

- Examination of complete blood cell count with differential;
- Blood tests: glucose, urea nitrogen, creatinine, electrolytes, transaminases, γGT, direct and indirect bilirubin, amylase, LDH, CPK, sieroprotein electrophoresis, hsPCR, total cholesterol and HDL;
- Pregnancy test at enrolment.

Virological evaluation

At screening and in case of virologic failure during the protocol, it will be performed a blood test to get 2 aliquots of 5x10^6 frozen viable cells in order to perform the sequence of the gp120 gene and subsequent determination of viral tropism by algorithm .

It will also be required, where available, plasma stored under appropriate conditions, from the patient when in a condition of detectable viremia (2 mL of plasma required to perform the test Trofile post-hoc).

At baseline and after 2, 4, 8 and 12 weeks after start of the study, and then at 4, 6 and 9 months from the beginning of the study and at the end of the period of administration of MVC:

- Quantification of HIV-RNA.

Immunological evaluations

At baseline and after 2, 4, 8, 12 weeks after start of the study, then at 4, 6 and 9 months from the beginning of the study and at the end of the period of administration of maraviroc:

- Immunophenotypic analysis (lymphocytes memory and naïve CD4 & CD8);

- Analysis of chemokine mRNA;

- Analysis of the levels of plasma IL-7/IL-7R.

The first parameter (chemokines mRNA) is particularly important since the blockage of cellular co-receptor such ad CCR5 could also influence the process of internalisation of β-chemokines. Phenotypes on the cell surface will be evaluated through cytofluorimetry (fluorescein isothiocyanate [FITC], phycoerytrin [PE], and phycoerytrin-cyanin 5 [PCy5]). CD45RA/CD62L/CD4, CD4/CD38, CD8/CD38 in order to measure naïve, memory, and activated CD4+ and CD8+ will be analyzed. The proliferation of T lymphocytes will be evaluated through the quantification of the expression of the Ki67 nuclear antigen in CD4+ and CD8+ cells (an antigen which is expressed during the proliferation phases of cell cycles).

The *ex-vivo* characterization of system IL-7/IL-7R homeostasis will be carried out as well.

If available, a sample before first-line HAART will be analyzed for tropism.

Sample size and statistical analysis

This will be a pilot controlled clinical trial.

Sample size: assuming a proportion of "responder" patients (those patients who achieve, in 2 consecutive measurements, CD4> 200 cells/µL and CD4 lymphocytes recovery > 25 % from baseline, maintaining HIV-RNA <50 copies/mL) of 5% in the control arm, it is expected an absolute increase δ = 20% in the arm of intensification with MVC (responder patients with maraviroc = 25%). We have fixed the study power 1-β = 80% and α = 0.05, thus it is necessary to randomize 50 patients per arm, taking into consideration any protocol violations/lost to follow-up. The threshold of 20% difference between treated with MVC or non-treated is compatible with a proof of efficacy indicated by immunological trials with antagonists of CCR5 co-receptor.

A ​​1:1 randomization in blocks stratified by center will be made.

For statistical analysis, depending on the indication, the following tests will be carried out: chi-square test according to Mantel-Haenszel test, Kruskal Wallis, Log-rank test and Kaplan-Meier analysis model multivariate Cox and logistic regression .

Comparison of CD4+ and CD8+ count between the study arms A and B and at the different time-points will be assessed by t-test for independent and dependent samples respectively in an intention to treat (ITT) analysis. Statistical evaluation of simple and composite endpoints will be analyzed by binary logistic regression models for the odds ratio estimation and the related 95% confidence intervals. Odds ratios of the MVC arm will be controlled in the regression models for CD4+ counts at baseline. Statistical significance level will be estimated by Wald test and was set at .05 for all of the analyses. In the per-protocol (PP) analysis, patients who dropped the study or switched treatment will be included until their last observation in the randomization arm.

Immunological parameters at baseline (T0), after 12 (w12) and 48 weeks (w48) of MVC intensification will be analyzed with GraphPad 5 PRISM software. Wilcoxon and Mann-Whitney U test will be used. All tests are 2-sided and differences will be considered statistically significant at p<0.05.

Withdrawal from the study

The motivation and the exit date from the study will have to be reported in the medical record of each patient.

The study participants are free to withdraw from the participation at any time, without any explanation and without any prejudice against any and/or future care that should be applied in the Clinical Center where they are followed.

Also, the study participants can be requested to withdraw from the study at any time at the discretion of the investigator, if participation in the study involves any kind of risk for the patient’s health.

**Data sources and medical records**

The investigator has the responsibility to preserve the study original data as well as the list of names and addresses of patients included and signed informed consents for 15 years.

For each included patient, an Italian medical records must be completed; its accuracy and reliability of compilation will be attested by the signature of the investigator in charge. Corrections are allowed deleting the wrong data with a line, and rewriting the correct side with the date and the countersignature of charge. Use of correction fluid is not allowed.

The investigator will ensure that the study participants have the proper training about the antiretrovirals and that any information relevant for the conduct of the study is notified to co-investigators involved.

**Costs**

The necessary medication for the study will be provided free of charge by Pfizer for the entire duration of the study protocol. The funding for the shipment of biological samples for immunological analysis planned as a secondary objective of the protocol, through funds dedicated to this research by the coordinating center will be also provided.

Bibliography

1. Lai JP, Yang JH, Douglas SD, Wang X, Riedel E, Ho WZ. Clin Diagn Lab Immunol 2003;10:1123-1128.
2. Wasmuth JC, Nischalke HD, Jütte A, et al. Antiviral Res 2004;61:207-212.
3. Marchetti G, Meroni L, Molteni G, et al. Antivir Ther 2004;9:447-452.
4. Sasson SC, Zaunders JJ, Zanetti G, et al. J Infect Dis 2006;193:505-514.
5. Al Harthi L, Marchetti G, Steffens C, et al. J Immunol Meth 2000;237:187-197.
6. Hazenberg M, Otto SA, Cohen Stuart J, et al. Nature Med 2000;6:1036-1042.
7. Cicala C, Arthos J, Martinelli E, et al. Proc Natl Acad Sci USA 2006;103:3746-3751.
8. Huang W, Eshleman SH, Toma J, et al. J Virol 2007;81:7885-7893.

Legend. mg: milligram; BID: bis in die; IL-7: interleukin7; IL-7R: interleukin7 receptor; µL: microliters; w: week; HAART: Highly Active Anti-Retroviral Therapy; EC/IRB: ethics committees/institutional review boards; cp/ml: copies/millilitres; ELISA: enzyme-linked immunosorbent assay; IL-2: interleukin2; α,β,γ IFN: α,β,γ interferon; G-CSF: Granulocyte colony-stimulating factor; GM-CSF: Granulocyte-macrophage colony-stimulating factor; COPD: Chronic obstructive pulmonary disease; SGOT: Serum Glutamic Oxaloacetic Transaminase; SGPT: Serum Glutamic Pyruvic Transaminase; ANC: Absolute Neutrophil Count; γGT: gamma glutamiltranspepsidase; LDH: L-lactate dehydrogenase; CPK: creatinephosphokinase; hsPCR:   high-sensitivity C reactive protein; HDL: High Density Lipoprotein
