# Supplementary material for: Maraviroc as Intensification Strategy in HIV-1 Positive Patients with Deficient Immunological Response: an Italian Randomized Clinical Trial
Source: PLoS One. 2013 Nov 14;8(11):e80157. doi: 10.1371/journal.pone.0080157 (PMC3828227; doi:10.1371/journal.pone.0080157)
Supplement: Protocol S2 — Protocol in Italian. (DOC) [file pone.0080157.s003.doc]

##### PROTOCOLLO DI STUDIO

##### Razionale

Il miglioramento sub-ottimale delle conte delle cellule CD4 non è raro nei pazienti infettati da HIV-1 che presentano un HIV-RNA plasmatico a livelli soppressi[1]. Una diminuzione nelle conte delle cellule CD4+ è stata osservata in pazienti con viremia a livelli bassi o del tutto soppressi.

Anche se l'efficacia dei farmaci antiretrovirali correnti è evidente, alcune combinazioni antivirali sono molto efficaci nella soppressione dell’HIV-RNA mentre non esercitano alcun effetto sulla ricostituzione del sistema immune. Sia l’attivazione dei linfociti T che la fibrosi del tessuto linfoide periferico potrebbero generare un ambiente biologico in cui è possibile che si verifichi una diminuzione delle conte delle cellule CD4+ in presenza di livelli di viremia plasmatica bassi o soppressi.

Un'altra ipotesi affascinante, che ha ancora essere completamente analizzata e spiegata, è che la ricostituzione dello pool esaurito di linfociti CD4+ è ostacolata da un eccesso di proteina HIV-1 gp120. Questa abbondante produzione potrebbe essere neutralizzata da un inibitore del co-recettore CCR5, che rappresenta uno degli mezzi principali di aggancio di HIV-1 alla cellula ospite.

Dal punto di vista clinico, un’analisi post-hoc dello studio MERIT, che confrontava la sicurezza e l’efficacia di Maraviroc versus Efavirenz in pazienti naive alla terapia antiretrovirale, ha mostrato un maggiore e più rapido incremento della conta dei CD4 nei pazienti che assumevano Maraviroc rispetto a quelli nel braccio di trattamento con Efavirenz e questo dato era statisticamente significativo; inoltre nel braccio di trattamento con Maraviroc una maggiore proporzione di pazienti con CD4 al baseline inferiore a 200 cellule/µL ha raggiunto o superato questa soglia. Anche per i pazienti che non hanno ottenuto la soppressione virologica sono stati osservati una minore probabilità di andare incontro ad eventi AIDS-definenti ed un incremento della conta dei CD4 [2]. L’incremento della conta dei CD4 nei pazienti trattati con Maraviroc versus placebo è stato osservato anche a diversi dosaggi di Maraviroc [3,4] e nei pazienti con ceppo virale Dual/Mixed o X4-tropico nonostante la mancata soppressione virologica [5, 6]. Si tratta però sempre di osservazioni fatte nell’ambito di studi con altri obiettivi primari, mentre non è stato eseguito fino ad ora uno studio che si prefigga l’obiettivo di verificare direttamente questa ipotesi.

Osservazioni analoghe sono state fatte in pazienti che assumevano enfuvirtide [7], ma un possibile uso di tale farmaco su più ampia scala in pazienti con successo virologico è stato probabilmente frenato soprattutto dalla scarsa tollerabilità del farmaco, in particolare per la sua somministrazione per via sottocutanea, causa di frequenti reazioni cutanee a livello del sito di iniezione. Abbiamo scelto Maraviroc per la sua maggiore maneggevolezza, che lo rende più adatto ad un uso per la sola intensificazione di uno schema terapeutico che ha già determinato il successo virologico.

Considerando tutto questo, vorremmo condurre una sperimentazione clinica controllata pilota che arruolerà una popolazione di pazienti infettati da HIV-1 che hanno raggiunto velocemente una soppressione virologica senza una ricostituzione del loro sistema immune. Il dosaggio scelto è quello standard utilizzato per il trattamento dell’infezione da HIV, tenendo conto delle modifiche da effettuarsi a seconda dei farmaci concomitanti come da scheda tecnica del prodotto, che è anche il dosaggio su cui abbiamo maggiori dati in termini di sicurezza ed efficacia. La durata di un anno è stata scelta in base al fatto che gli incrementi di CD4 sono stati osservati negli studi clinici precedentemente segnalati a 48 settimane di trattamento, la durata scelta dovrebbe essere quindi sufficiente perché l’effetto desiderato possa essere evidenziato.

1. F. Aiuti, I. Mezzaroma, Failure to reconstitute CD4+ T-cells despite suppression of HIV replication under HAART, AIDS Rev. 8(2006) 88–97.
2. A Lazzarin, M Battegay, DA Cooper, and others. CD4+ cell restoration at 48weeks in the maraviroc (MVC) treatment-naive (TN) MERIT trial. 48th International Conference on Antimicrobial Agents and Chemotherapy (ICAAC 2008). Washington, DC. October 25-28, 2008. Abstract H-1248
3. Hardy D, Reynes J, Konourina I, Wheeler, Moreno S, van der Ryst E, *et al.* Efficacy and safety of Maraviroc plus optimized background therapy in treatment-experienced patients infected with CCR5-tropic HIV-1: 48-week combined analysis of the MOTIVATE studies [abstract #792]. In: *15th Conference on Retroviruses and Opportunistic Infections*; 3-6 February 2008; Boston, Massachusetts; 2008
4. D. Asmuth, J. Goodrich, D. Cooper, R. Haubrich, N. Rajicic, H. Valdez, B. Hirschel, H. Mayer, CD4+ cell restoration after 48 weeks in the maraviroc (MVC) treatment-experienced (TE) trials MOTIVATE 1 and 2 Abstract TUPE0050 17^th^ International AIDS conference, 3-8 August 2008, Mexico City
5. Mayer H, van der Ryst E, Saag M, Clotet B, Fatkenheuer G, Clumeck N, *et al.* Safety and efficacy of maraviroc, a novel CCR5 antagonist, when used in combination with optimized background therapy for the treatment of antiretroviral-experienced subjects with dual/mixed-tropic HIV-1: 24-week results of a phase 2b exploratory trial [abstract #THLB0215]. In: *XVI International AIDS Conference*; 13-18 August 2006; Toronto, Canada; 2006
6. Gerd Fätkenheuer, Mark Nelson, Adriano Lazzarin, Irina Konourina, Andy I.M. Hoepelman, Ph.D., Harry Lampiris, Bernard Hirschel, Pablo Tebas, François Raffi, Benoit Trottier, Nicholaos Bellos, Michael Saag, David A. Cooper, Mike Westby, Margaret Tawadrous, John F. Sullivan, Caroline Ridgway, Michael W. Dunne, Steve Felstead, Howard Mayer, and Elna van der Ryst, for the MOTIVATE 1 and MOTIVATE 2 Study Teams, Subgroup Analyses of Maraviroc in Previously Treated R5 HIV-1 Infection, N Engl J Med 2008;359:1442-55.
7. Svicher V, Aquaro S, D'Arrigo R, Artese A, Dimonte S, Alcaro S, Santoro MM, Di Perri G, Caputo SL, Bellagamba R, Zaccarelli M, Visco-Comandini U, Antinori A, Narciso P, Ceccherini-Silberstein F, Perno CF, [Specific enfuvirtide-associated mutational pathways in HIV-1 Gp41 are significantly correlated with an increase in CD4(+) cell count, despite virological failure.](http://www.ncbi.nlm.nih.gov/pubmed/18419549?ordinalpos=4&itool=EntrezSystem2.PEntrez.Pubmed.Pubmed_ResultsPanel.Pubmed_DefaultReportPanel.Pubmed_RVDocSum) J Infect Dis. 2008 May 15;197(10):1408-18.

##### Obiettivi

Gli obiettivi primari dello studio sono:

- valutare **l'efficacia clinica (in termini di protezione dall’insorgenza di patologie opportunistiche) e immunologica** del trattamento di intensificazione della HAART con Maraviroc (MVC), nel trattamento dell’infezione da HIV-1 in pazienti con linfociti CD4+ ≤200 cellule/μL e/o con recupero dei linfociti ≤25% e completa soppressione della carica virale dopo 12 mesi di terapia antiretrovirale. I pazienti potranno essere arruolati anche se il profilo dei propri CD4 è stabile senza alcun miglioramento, con un valore assoluto intorno alle 200 cellule/μL.
- valutare gli effetti della intensificazione della HAART con Maraviroc (MVC) sui **parametri immunologici e virologici**.
- valutare la **tollerabilità** della intensificazione della HAART con Maraviroc (MVC) e l’insorgenza di effetti collaterali correlati al farmaco.

Gli obiettivi secondari dello studio sono:

##### valutare l’effetto del trattamento di intensificazione della HAART con Maraviroc (MVC) sulla modificazione dei seguenti parametri immunologici:

analisi dell’mRNA delle chemiochine, linfociti CD4 helper memory e naïve, sistema omeostatico IL-7/IL-7R, “T-cell receptor excision circles” (TREC).

##### Endpoints

Sono definiti “responders” al trattamento I pazienti che ottengono il raggiungimento, in 2 misurazioni consecutive, di CD4 >200 cellule/μL e recupero dei linfociti CD4 >25% dal baseline, mantenendo un valore di HIV-RNA <50 copie/mL.

End-point primario:

- variazione dall’arruolamento (baseline) nella conta assoluta e nella percentuale dei linfociti T CD4+, a 3 e a 12 mesi dal baseline; sarà valutato il raggiungimento, in 2 misurazioni consecutive, di CD4 >200 cellule/μL e recupero dei linfociti CD4 >25%, mantenendo un valore di HIV-RNA <50 copie/mL.

End-point secondari:

- riduzione della proporzione dei pazienti con comparsa di eventi clinici HIV-correlati.
- proporzione dei pazienti con eventi avversi gravi (WHO III-IV).

**Disegno dello studio**

Studio randomizzato, in aperto e multicentrico che valuterà l’intensificazione della HAART come trattamento dei pazienti con infezione da HIV-1 tramite la terapia con Maraviroc (MVC) nel trattamento dell’infezione da HIV-1 in pazienti con conta di linfociti CD4+ ≤200 cellule/μL e/o con recupero del numero di linfociti CD4+ ≤25% rispetto all’inizio della HAART e con completa soppressione della carica virale dopo 12 mesi di HAART. I pazienti potranno essere arruolati anche se il profilo dei propri CD4 è stabile senza alcun miglioramento, con un valore assoluto intorno alle 200 cellule/μL.

**Bracci dello studio**

I pazienti arruolati nello studio (100 in totale) saranno assegnati casualmente a due bracci uguali per numerosità, un gruppo riceverà Maraviroc (MVC) come intensificazione della HAART e l’altro gruppo continuerà la propria HAART senza alcuna intensificazione.

**Schema di trattamento**

Lo schema posologico del Maraviroc (MVC) sono: da 150mg due volte al giorno a 600mg due volte al giorno, a seconda degli altri farmaci antiretrovirali che il paziente sta già assumendo. Maraviroc (MVC) verrà somministrato al dosaggio di 150mg due volte al giorno insieme a nelfinavir e agli inibitori della proteasi potenziati farmacologicamente da ritonavir (PI/rtv), quali atazanavir/rtv, darunavir/rtv, lopinavir/rtv e saquinavir/rtv; 300mg due volte al giorno con nevirapina, fosamprenavir/rtv, tipranavir/rtv, raltegravir ed enfuvirtide (T-20); 600mg due volte al giorno con efavirenz ed etravirina (TMC125). Nel caso della somministrazione all’interno di un regime terapeutico comprendente un PI/rtv (atazanavir/rtv, darunavir/rtv, lopinavir/rtv e saquinavir/rtv), il dosaggio di Maraviroc (MVC) sarà sempre di 150mg due volte al giorno.

**Casistica**

E’ previsto l’arruolamento di 100 pazienti consecutivamente osservati, con le caratteristiche succitate, presso il Dipartimento di Scienze Cliniche, Sezione di Malattie Infettive ed Immunopatologia dell’Università degli Studi, Ospedale “Luigi Sacco”, Milano, a partire da giugno 2008.

Gli altri Centri clinici che parteciperanno allo studio sono: Clinica delle Malattie Infettive, Ospedale San Paolo: Prof.ssa Antonella d’Arminio Monforte; 1^a^+2^a^ Divisioni di Malattie Infettive, Ospedale Luigi Sacco, Milano: Dr. Giuliano Rizzardini; Clinica delle Malattie Infettive, Istituto Scientifico H San Raffaele, Milano: Prof. Adriano Lazzarin; Clinica delle Malattie Infettive, Università di Brescia: Prof. Giampiero Carosi; Clinica delle Malattie Infettive, Università di Tor Vergata, Roma: Prof. Massimo Andreoni; Clinica delle Malattie Infettive, Università di Torino: Prof. Gianni Di Perri; Istituto Nazionale per le Malattie Infettive, Ospedale L. Spallanzani, III Divisione Malattie Infettive, Roma: Dr. Andrea Antinori; Clinica delle Malattie Infettive, Ospedale San Gerardo, Monza: Dr. Andrea Gori; Centro Emofilia – Ospedale Maggiore Policlinico, Milano: Dr.ssa Myrvet Muça Perja; Istituto Nazionale per le Malattie Infettive, Ospedale L. Spallanzani, IV Divisione Malattie Infettive, Roma: Dr. Pasquale Narciso; Divisione di Malattie Infettive, Ospedale “S. Maria Goretti”, Latina: Prof. Claudio Mastroianni; Divisione Dipartimento Urgenze Infettivologiche ad Alta Complessità e correlate all'AIDS, Ospedale Cotugno, Napoli: Dr. Antonio Chirianni; U.O. Malattie Infettive, Ospedale S. Spirito, Pescara: Dr. Giustino Parruti; Clinica di Malattie Infettive, Policlinico di Bari: Prof. Giuseppe Pastore; Div. Malattie Infettive, Ospedale di Circolo, Busto Arsizio: Dr.ssa Tiziana Quirino; Div. Malattie Infettive, Ospedali Riuniti, Bergamo: Dr. Franco Maggiolo; Clinica Malattie Infettive, Ospedale San Martino, Genova: Prof. Claudio Viscoli; Div. Malattie Infettive, Ospedale S. Maria Annunziata, Antella - Firenze: Dr. Francesco Mazzotta; Servizio Regionale di Immunologia Clinica e tipizzazione tissutale, Azienda Ospedaliero-Universitaria, Torrette di Ancona: Dr. Luca Butini; Div. A di Malattie Infettive, Ospedale Amedeo di Savoia, Torino: Dr. Pietro Caramello; Istituto Clinica delle Malattie Infettive, Università Cattolica del Sacro Cuore, Roma: Prof. Roberto Cauda; Clinica Malattie Infettive, Policlinico Universitario, Modena: Dr.ssa Cristina Mussini; U.O. Malattie Infettive, Policlinico S. Matteo, Pavia: Dr. Renato Maserati; U.O. Malattie Infettive, Azienda Policlinico Umberto I, Roma: Prof. Vincenzo Vullo; Clinica delle Malattie Infettive, Policlinico Monteluce, Perugia: Prof. Franco Baldelli; Clinica Malattie Infettive e Tropicali, Policlinico S. Matteo, Pavia: Prof. Gaetano Filice.

Verranno ammessi allo studio 100 pazienti HIV positivi con conta di linfociti CD4+ ≤200 cellule/μL e/o con un recupero nella conta dei linfociti CD4+ ≤25%, con carica virale soppressa (HIV-RNA ≤50 copie/mL) dopo 12 mesi di trattamento con farmaci antiretrovirali. Potranno anche essere arruolati pazienti con stabile profilo dei propri CD4 senza alcun miglioramento, con un valore assoluto intorno alle 200 cellule/μL.

##### Durata dello studio

Sono previsti 6 mesi per l’arruolamento dei pazienti e 12 mesi di somministrazione di Maraviroc (MVC).

Criteri di inclusione:

- maschi o femmine di età superiore ai 18 anni;

- positività sierologica per HIV accertata con metodica ELISA e confermata da Western Blot;
- pazienti con linfociti T CD4+ ≤200 cellule/μL e/o con un recupero nella conta dei linfociti CD4+ ≤25% dopo trattamento con antiretrovirali da almeno 12 mesi; pazienti con stabile profilo dei propri CD4, senza alcun miglioramento, con un valore assoluto intorno alle 200 cellule/μL;
- pazienti con carica virale ≤50 copie/mL dopo trattamento con antiretrovirali da almeno 12 mesi;
- pazienti con test di gravidanza negativo almeno 14 giorni prima dell’inizio del trattamento. Tutti i pazienti dovranno garantire l’utilizzo di efficaci metodiche anticoncezionali nel periodo compreso tra 4 settimane prima e 8 dopo l’inizio del trattamento;
- comprensione e sottoscrizione del consenso informato scritto.

Criteri di esclusione:

- allergia/intolleranza nota ai farmaci in studio;
- trattamento effettuato entro l’anno dal potenziale screening con farmaci immunomodulanti o fattori di crescita o citochine (IL-2, α-, β-, γ-IFN, G-CSF, GM-CSF);
- pazienti affetti da patologie opportunistiche o neoplasie in atto;
- pazienti affetti da patologie cardiovascolari e/o anomalie elettrocardiografiche;
- pazienti affetti da patologie respiratorie, quali asma, broncopneumopatie croniche ostruttive e restrittive;
- trattamento sistemico con corticosteroidi entro 4 settimane dall’inizio del trattamento;
- pazienti con sospetta o accertata malattia autoimmune o infiammatoria cronica;
- tossicodipendenti attivi, etilisti e pazienti ritenuti dall’investigatore scarsamente collaboranti;
- pazienti con i seguenti parametri di laboratorio determinati 2 settimane prima dell’inizio del trattamento:
- SGOT e SGPT ≥2,5 volte il limite superiore della norma;
- creatinina sierica >1,5 volte il limite superiore della norma;
- ANC <1000/μL;
- emoglobina <10 g/dL;
- piastrine <75.000/μL;
- reticolociti >2%;
- indice di Karnofsky <50.

### Metodologia dello studio

Valutazione clinica.

Al momento dell’arruolamento, alle settimane 2, 4, 8, 12 dall’inizio dello studio, in seguito ai mesi 4, 6 e 9 dall’inizio dello studio e alla fine del periodo di somministrazione di Maraviroc (MVC):

- Anamnesi
- Esame obiettivo
- Valutazione dell’indice di Karnofsky

Esami ematochimici.

Al momento dell’arruolamento e successivamente alle settimane 2, 4, 8, 12 dall’inizio dello studio, in seguito ai mesi 4, 6 e 9 dall’inizio dello studio e alla fine del periodo di somministrazione di Maraviroc (MVC):

- Esame ematologico completo con formula leucocitaria
- Esami ematochimici: glicemia, azotemia, creatininemia, elettroliti, transaminasi, γGT, bilirubina diretta ed indiretta, amilasemia, LDH, CPK, elettroforesi sieroproteica, hsPCR, colesterolo totale e HDL.
- Test di gravidanza all’arruolamento

Valutazione virologica

Allo screening e in caso di fallimento virologico durante il protocollo, verrà eseguito un prelievo di sangue per ottenere 2 aliquote da 5 X 10^6 cellule vitali congelate al fine di eseguire un test di sequenza del gene *gp120* e successiva determinazione del tropismo virale mediante algoritmo.

Sarà inoltre richiesto, ove disponibile, plasma opportunamente conservato, proveniente dal paziente quando in condizione di viremia detettabile (necessari 2 mL di plasma per eseguire il test Trafile post-hoc).

Al momento dell’arruolamento e dopo 2, 4, 8, 12 settimane dall’inizio dello studio, in seguito ai mesi 4, 6 e 9 dall’inizio dello studio e alla fine del periodo di somministrazione di Maraviroc (MVC):

- quantificazione dell’HIV-RNA attraverso branched-DNA (b-DNA).

# Valutazione immunologica

Al momento dell’arruolamento e dopo 2, 4, 8, 12 settimane dall’inizio dello studio, in seguito ai mesi 4, 6 e 9 dall’inizio dello studio e alla fine del periodo di somministrazione di Maraviroc (MVC):

- Analisi immunofenotipica (linfociti memory e naïve CD4 & CD8);
- Analisi dell’mRNA delle chemiochine;
- Analisi dei livelli di IL-7/IL-7R plasmatica;
- Analisi della funzionalità timica (TRECs).

#### Dimensione del campione ed analisi dei dati

Sperimentazione clinica controllata pilota.

Dimensione del campione: ipotizzando una proporzione di pazienti “responder” (intendendo come tali, come già specificato in precedenza, i pazienti che ottengono il raggiungimento, in 2 misurazioni consecutive, di CD4 >200 cellule/μL e recupero dei linfociti CD4 >25% dal baseline, mantenendo un valore di HIV-RNA <50 copie/mL) pari al 5% nel braccio dei controlli, è atteso un incremento assoluto δ = 20% nel braccio dei pazienti trattati con Maraviroc (*pazienti responder in maraviroc=25%*); fissati potenza 1-β=80% e α=0.05, si rende necessario randomizzare 50 pazienti per braccio, tenendo in considerazione eventuali violazioni di protocollo / persi al follow-up. La soglia del 20% di differenza tra trattati con MVC o non-trattati è compatibile con una prova di efficacia immunologica indicata da trials con antagonisti del co-recettore CCR5.

Verrà operata una randomizzazione (1:1) a blocchi stratificata per centro.

Per le analisi statistiche, a seconda delle indicazioni, verranno eseguiti principalmente i seguenti test: test del chi-quadrato secondo Mantel-Haenszel, Kruskal Wallis, Log-Rank test e curve di Kaplan-Meier, modello di analisi multivariata secondo Cox e regressione logistica.

##### Uscita dallo studio

La motivazione e la data dell’uscita dallo studio dovranno essere segnalate sulla cartella clinica di ogni paziente.

I soggetti partecipanti allo studio sono liberi di recedere da tale partecipazione in ogni momento senza dovere alcuna spiegazione e senza pregiudizio alcuno nei confronti di eventuali e/o future cure a cui dovessero ricorrere nel Centro Clinico presso cui vengono seguiti.

Inoltre i soggetti possono essere fatti uscire dallo studio in qualunque momento a discrezione del medico sperimentatore, se la partecipazione allo studio rappresenta un rischio di qualsiasi natura per la salute del paziente.

**Dichiarazione di Helsinki e comitato etico**

Lo studio verrà condotto in accordo con i principi della dichiarazione di Helsinki. Il protocollo ed il foglio informativo/consenso informato per il paziente dovranno essere approvati dal Comitato Etico di ogni Centro Clinico prima che lo studio inizi ad arruolare pazienti afferenti al Centro Clinico stesso.

Il medico sperimentatore ha la responsabilità di informare il proprio Comitato Etico di ogni Evento Avverso e di ogni emendamento al protocollo secondo quanto previsto dai decreti legislativi correnti tenendone copia nei propri archivi.

**Consenso informato dei pazienti**

Il medico sperimentatore ha la responsabilità di dare al paziente una adeguata informazione circa gli scopi, i benefici ed i possibili rischi dello studio. Il medico sperimentatore è inoltre responsabile di ottenere il consenso scritto del paziente prima di includerlo nello studio (presentato separatamente al Comitato Etico).

##### Fonti dei dati e cartelle cliniche

Lo sperimentatore ha la responsabilità di conservare i dati in originale relativi allo studio nonchè la lista dei nomi e degli indirizzi dei pazienti inclusi ed il consenso informato firmato per 15 anni.

Per ogni paziente incluso dovrà essere compilata in italiano una cartella clinica la cui accuratezza ed attendibilità di compilazione saranno attestate dalla firma dello sperimentatore responsabile. Correzioni cancellando con una linea il dato sbagliato e riscrivendo il valore corretto accanto con la data e la controfirma del responsabile. Non è ammesso l'uso di correttori fluidi.

Lo sperimentatore responsabile garantirà che i partecipanti allo studio abbiano l'adeguato addestramento e che ogni informazione rilevante per la buona riuscita dello studio sia comunicata ai co-investigatori coinvolti.

##### Costi

Il farmaco necessario per lo studio verrà fornito a titolo gratuito da Pfizer per l’intera durata del protocollo di studio. E’ inoltre prevista la copertura finanziaria per la spedizione dei campioni per le fini analisi immunologiche previste come obiettivo secondario del protocollo tramite fondi dedicati a questa ricerca a cura del centro coordinatore.

Legend: HAART: Highly Active Anti-Retroviral Therapy; µL: microliters; IL-7: interleukin7; IL-7R: interleukin7 receptor; ml: milliliters; mg: milligram; ELISA: enzyme-linked immunosorbent assay; IL-2: interleukin2; α,β,γ IFN: α,β,γ interferon; G-CSF: Granulocyte colony-stimulating factor; GM-CSF: Granulocyte-macrophage colony-stimulating factor; COPD: Chronic obstructive pulmonary disease; SGOT: Serum Glutamic Oxaloacetic Transaminase; SGPT: Serum Glutamic Pyruvic Transaminase; ANC: Absolute Neutrophil Count; γGT: gamma glutamiltranspepsidase; LDH: L-lactate dehydrogenase; CPK: creatinephosphokinase; hsPCR:   high-sensitivity C reactive protein; HDL: High Density Lipoprotein; TRECs: T-cell receptor excision circles.
