# Supplementary material for: Maraviroc as Intensification Strategy in HIV-1 Positive Patients with Deficient Immunological Response: an Italian Randomized Clinical Trial
Source: PLoS One. 2013 Nov 14;8(11):e80157. doi: 10.1371/journal.pone.0080157 (PMC3828227; doi:10.1371/journal.pone.0080157)
Supplement: Text S1 — EC/IRBs. (DOC) [file pone.0080157.s004.doc]

**Ethics committees/institutional review boards that were involved in the exam and approval of the NCT 00884858 protocol at various clinical centers.**

The ethics committees/institutional review boards of the following Infectious Diseases units approved the study protocol: Divisione Clinicizzata di Malattie Infettive, DIBIC “Luigi Sacco”, Università degli Studi, Milano, Divisione di Malattie Infettive, Ospedale Luigi Sacco, Milano, II Divisione di Malattie Infettive, Ospedale Luigi Sacco, Milano, and Divisione di Malattie Infettive, Ospedale di Circolo, Busto Arsizio, VA; Clinica di Malattie Infettive e Tropicali, Università degli Studi, Brescia; Clinica delle Malattie Infettive, Ospedale Amedeo di Savoia, Università degli Studi, Torino, and Divisione A di Malattie Infettive, Ospedale Amedeo di Savoia, Torino; Clinica di Malattie Infettive, A.O.-Universitaria Policlinico, Bari; Clinica delle Malattie Infettive, Ospedale San Martino, Università degli Studi, Genova; Divisione di Malattie Infettive, Ospedale S. Maria Annunziata, Antella, Firenze; Servizio di Immunologia Clinica e Tipizzazione. Tissutale, A.O.-Universitaria, Torrette di Ancona; Istituto di Clinica delle Malattie Infettive, Università Cattolica del Sacro Cuore, Roma; U.O. Malattie Infettive, Università La Sapienza, Policlinico Umberto I, Roma; Clinica delle Malattie Infettive, Policlinico Monteluce, Perugia; Divisione Clinicizzata di Malattie Infettive, Ospedale Santo Spirito, Pescara; Divisione Clinicizzata di Malattie Infettive, Ospedale San Gerardo, Monza; Clinica delle Malattie Infettive, Policlinico "Tor Vergata", Roma; III Divisione di Malattie Infettive I.N.M.I “Lazzaro Spallanzani”, Roma, and IV Divisione di Malattie Infettive I.N.M.I “Lazzaro Spallanzani”, Roma; Clinica delle Malattie Infettive, Dipartimento di Scienze della Salute, Polo Universitario San Paolo, Università degli Studi, Milano, Italy.

Legend: DIBIC: Dipartimento di Scienze Biomediche e Cliniche; VA: Varese; A.O: Azienda Ospedaliera; U.O. Unità Operativa; I.N.M.I: Istituto Nazionale per le Malattie Infettive.
